# Effects of self- and partner’s online disclosure on relationship intimacy and satisfaction

**DOI:** 10.1371/journal.pone.0212186

**Published:** 2019-03-04

**Authors:** Juwon Lee, Omri Gillath, Andrew Miller

**Affiliations:** 1 Department of Psychology, Dietrich College of Humanities and Social Sciences, Carnegie Mellon University, Pittsburgh, Pennsylvania, United States of America; 2 Department of Psychology, College of Liberal Arts & Sciences, University of Kansas, Lawrence, Kansas, United States of America; 3 School of Medicine, University of Kansas Medical Center, Kansas City, Kansas, United States of America; University of Auckland, NEW ZEALAND

## Abstract

Most research on the effects of disclosure on close relationships have been done using offline disclosure. However, disclosure done online has disparate features and thus its effects on relationships may also differ. In five studies and using primes emulating Facebook timelines and messages, we compared the effects of disclosure depth on intimacy and satisfaction in online vs. offline contexts, in romantic vs. friend relationships, and with differing content (self- vs. partner-focused). After demonstrating consistent differences, we examined one mechanism that accounted for the differential effects of online vs. offline disclosure in romantic relationships: perceived inclusivity of the recipients. Results revealed that greater disclosure was associated with higher relational intimacy and satisfaction when done offline (Studies 1 and 4), and lower intimacy and satisfaction when done online (Studies 1–4), in both the discloser (Study 1) and his or her partner (Studies 2–4). The negative association between online disclosure and intimacy was present in romantic relationships, but not in friendships (Study 1). Importantly, this effect only appeared when perceived inclusivity of recipients was high (Study 4). Focusing the online disclosure content on the partner/relationship dissipated its negative effects (Study 5). Together, these studies extend further knowledge on how the effects of disclosure are contextualized, and suggest that disclosure done publicly online may be detrimental to romantic relationships.

## Introduction

In today’s modern world, with the ubiquity of the Internet [1] and the immense upsurge in the availability and usage of communication technology, many social interactions and even full-blown relationships are taking place partially or completely online. For example, people are increasingly using the Internet to initiate [2], maintain [3], and even terminate [4] romantic relationships. Hence, many relational processes are taking place online, including self-disclosure—the sharing of information about oneself with another person [5] (for the sake of brevity, we refer to self-disclosure as “disclosure” in the document). Online social networking platforms, such as Facebook, Twitter, and Instagram have provided grounds for and encouraged the growth of online disclosure. The growth reflects both in *breadth*—the amount of information, time spent communicating, and topic variety, and in *depth*—the relevance of the topic to the innermost self, with some topics being more personal than others [6–8]. Because of this, understanding the role online disclosure plays in relationships is highly important.

Despite the massive increase of relational processes taking place online and increase in scholary interest, most of the research on relational processes and their effects on relationships has been done in regard to offline interactions. This may have hindered the generalizability of existing findings, potentially lowering their relevance to today’s relationships. Although some findings may be similar across online and offline contexts, it is also likely that a relational process unfolds differently depending on the setting or the context [9]. These contextual differences may affect the interpretations of the disclosure and in turn yield different relational outcomes.

Online disclosure has different characteristics from offline disclosure, mainly due to differences in *structural features of the message medium* [10]. For example, Facebook and Twitter easily allow and foster people to disclosure information in a way that is accessible to many recipients [11–13]. Further, this disclosure can include a lot of information [14] and does not highlight the need to get feedback from each of the recipients (less conversational) [15]. Conversely, offline disclosure is often done in a dyadic (one-to-one) context [16], and a response, verbal or non-verbal, from the recipient is usually expected [17].

The role disclosure plays in relational processes has been commonly theorized and studied as an offline dyadic process. For example, the two major models of disclosure and relational outcomes, Chelune and colleagues’ *person-situation interactional model* [18] and Reis and Shaver’s [19] *interpersonal process of intimacy model*, both focus on the associations between high-depth disclosure and intimacy (defined as *feelings of closeness and connectedness*) [20] in offline relationships. Moreover, their discussion of contextual factors (such as offline vs. online) is rather limited. Consequently, these models do not thoroughly account for the impact context may have on the dyadic process of disclosure. Below we briefly discuss the two models and needed adjustments due to shifts to online contexts.

The person-situation interactional model [18] focuses on the effect of disclosure on the recipient’s intimacy in the relationship. According to the model, once a disclosure is made, the recipient engages in interpreting the message’s intended meaning. This interpretation is based on various factors such as the disposition of the discloser and/or physical setting. For example, one would be more surprised to receive disclosure about romantic problems during a business meeting compared to during a coffee date between friends. These relational expectations and attributions are combined with the message content to affect intimacy. If the recipient’s interpretation of the message fits with the discloser’s intention, then the recipient’s intimacy is likely to increase. However, if the interpretation is different from what the discloser intended, this may negatively affect the recipient’s intimacy.

The interpersonal process of intimacy model [19] also describes the effects of disclosure on intimacy in the relationship; however it focuses on the effects of disclosure on the discloser’s intimacy. In the model, both the discloser’s and recipient’s interpretations play a role in a dynamic process that consists of several stages: An individual discloses personal information to the recipient; the information is then interpreted by the recipient based on the recipient’s disposition and situational features (an *interpretive filter*); the recipient then provides a response. If this response is interpreted by the discloser’s interpretive filter as understanding, validating, and caring (*high perceived responsiveness*), it leads to higher intimacy in the discloser [19, 21].

Although both of these models discuss the possible influence of contextual factors on the discloser’s and recipient’s intimacy, they do not delve further into which specific contextual factors may have an effect, and the particular mechanisms that underlie any differential effects of various disclosure contexts. Rather, the models focus on describing the *outcomes* of supposed contextual factors, such as the fit of the discloser’s intention and recipient’s interpretation [18] or high perceived responsiveness [19].

Without discerning the influence of different contexts on disclosure in relationships, it will be difficult to paint a full picture on how recent technological changes (i.e., online platforms) [22] are affecting relationships. Hence, we argue that existing models should be updated and extended to more fully account for the effects of context on disclosure. A more comprehensive account of disclosure should describe what attributes of the context interact with various stages of the disclosure process [19] to influence relational outcomes, and what mechanisms might explain these moderating effects.

In the current paper, we sought to identify various contextual factors of disclosure (such as online vs. offline, different relationship types, and varied content) and examine how variations in those factors differentially affect relational outcomes, namely relational intimacy and satisfaction (*the degree of overall positive experience in people’s interpersonal relationships*) [23]. We also examined one potential underlying mechanism—*inclusivity of recipients*—which could directly account for differential effects of disclosure on relational outcomes. We later further discuss how existing models of disclosure and relational outcomes may be expanded to more thoroughly explain the effects of different types of disclosure on relationships.

### Online vs. offline disclosure

With the many online platforms available for disclosure of personal information, and the high usage of these platforms, more and more people are disclosing online [7–8]. Online disclosure can occur through various mediums, such as emails, text messages, online forums, online social networks, in-game chats, and online classes. As we cannot examine all the ways online disclosure occurs, we focus here on an example of an online disclosure that is relevant to a broad population which uses online social networks (in contrast to, for example, only gamers or members of a specific forum). The example—disclosing on online SNS such as Facebook or Twitter—has a one-to-many format (*masspersonal communication*) [24]. We concentrated on this type of disclosure because it is currently relevant to many people and increasing exponentially in its usage [8, 25–26]. Furthermore, Facebook has captured an important spot in research, with 6,090,000 search results of “Facebook” on Google Scholar as of May 2018, and previous findings show this type of disclosure to have numerous outcomes for individuals [27–28] and relationships [29–30]. We compare this online disclosure to offline (face-to-face) dyadic disclosure, as offline disclosure has been the most researched in its processes and outcomes in a dyadic form (for a review see [31]). We are aware these two types of disclosure differ in many ways on top of one being done online and the other offline, such as being inclusive vs. exclusive, and occurring in a dyad vs. group. We elaborate on these differences in the general discussion.

By and large, the depth of offline disclosure has been shown to relate with many positive relational outcomes, such as liking [32], intimacy [21, 33], and satisfaction levels [34, 35] in close relationships (for a review see [31]). The findings on online disclosure and relational outcomes, however, are less conclusive. Some studies that investigated specific kinds of online disclosure show that it is related to positive relational outcomes. For example, Saslow and colleagues [30] showed that participants who included their partner in their Facebook profile picture exhibited higher relationship satisfaction compared to those who did not include the partner in their profile picture. Likewise, Papp and colleagues [29] found that participants who disclosed their romantic relationship status in their Facebook profile showed higher relationship satisfaction compared to those who did not exhibit their status. Toma and Choi [36] also found that participants who engaged in more Facebook self-presentational cues involving the relationship such as disclosing one’s romantic relationship status, writing on their partner’s Facebook page, and posting dyadic photographs increased in relationship commitment over time.

A number of other studies, however, have found online disclosure and related constructs to be associated with negative relational outcomes. For example, Papp and colleagues [29] found that disagreement between partners regarding their relationship status on their Facebook profiles results in tension, which in turn associated with lower relationship satisfaction in the female partner. Studies about online social network usage have found a mix of self- and perceived partner- associations with negative emotions [37], infidelity and breakup [38–39], and lower intimacy and satisfaction [40]. In addition, Zhao and colleagues [41] propose romantic relationships may face challenges because online social networks are settings in which multiple relationship goals (friendships, romantic relationships) interact with personal needs as people manage their presence online. Because the audience is both varied (friends, romantic partner, colleagues, etc.) and seem continuously present (“*context collapse*” [42]), it becomes difficult to cater self-presentations to one type of audience, and this can lead to tensions with the partner when expectations regarding relational self-presentation aren’t met. Together these studies depict an inconsistent set of findings, leaving the unanswered question of whether disclosure done online will have positive or negative outcomes for romantic relationships.

The literature on disclosure proposes that the *depth* of disclosure matters for relational outcomes more than the *breadth* of the disclosure [19]. Indeed, studies focusing on offline disclosure have provided evidence supporting this idea, showing that disclosure depth, rather than breadth, accounted for differences in relational outcomes such as liking [43]. When it comes to online disclosure, however, most studies have focused on the breadth of online disclosure. To our knowledge, no study has examined how differences in the depth of disclosure online associates with relational outcomes. In other words, it is unclear if disclosure depth online associates with relational outcomes in the same way as it does offline. Therefore, in the current paper we have focused on comparing the association between disclosure depth and relational outcomes in online vs offline disclosure, which is our first research question (RQ):

RQ1: Does online disclosure depth predict relationship intimacy and satisfaction differently from offline disclosure depth?

As any meaningful effect of the type of disclosure on relationships should exhibit itself not only in the discloser’s relational outcomes, but also the recipient/partner’s [18–19], we looked into both. We focused on the relational outcomes of intimacy and satisfaction because of their significance in relationship research [44–46] and implications for both personal outcomes, such as stress [47] and happiness [48], and relational outcomes, such as stability [49] and dissolution [50]. Moreover, intimacy and satisfaction can be seen to reflect different stages of a relationship. Intimacy is more relevant to the initiation and development [51–52] of a relationship, whereas satisfaction is more pertinent to its maintenance and longevity [49]. Assessing both these outcomes provides more information on what is important for different relational stages.

### Interactions between contexts: Online vs. offline, type of relationship, content

Although conditional effects of contextual factors on the disclosure process and relational outcomes have been presumed and studied (for a review see [53]), there are not many studies that have systematically investigated those effects, and likewise previous models of disclosure and relational outcomes [18–19] do not elaborate on contextual interactions. However, the burgeoning of online contexts for disclosure will increase the occurrence of any contextual effects and their interactions. Social interactions outside of the laboratory usually include multiple contexts rather than cleanly containing one. For example, if you are communicating with your partner online, there are at least two contexts: Online and romantic relationship. This emphasizes the importance of examining possible contextual effects and extending prior models of disclosure and relational outcomes to include more fine-tuned considerations of them. Disentangling the ways that these contexts (online/offline and relationship type) influence the disclosure process is important in order to gain a more complete picture of how disclosure affects relationships. Our second research question focuses on this contextual interaction:

RQ2: Does the association between online disclosure depth and relationship intimacy and satisfaction depend on the type of relationship?

The inconclusive and mixed findings on the association between online disclosure and relationship quality may be due to the lack of interest on processes and mechanisms that underlie that association [54]. As such, it is important to identify the broader mechanisms that underlie contextual effects or interactions. Additionally, this will enable relational outcomes to be predicted with any combination or variation of contexts, rather than be predicted only under specific situations. To our knowledge, no study so far has uncovered any mechanisms underlying the effects of online disclosure on relationships. We tested for and identified one mechanism that accounted for the differential effect of online disclosure on relational outcomes: inclusivity of recipients.

RQ3: Does inclusivity of the recipients account for the effect of online disclosure depth on romantic relationships?

Lastly, we wanted to see if any effect of online disclosure on relationships could be attenuated by testing another contextual interaction, the content of disclosure.

RQ4: Can the effect of online disclosure depth on romantic relationships be mitigated by differing the content of disclosure?

In whole, delineating contextual interactions and inherent mechanisms that affect the association between disclosure and relational outcomes will portray a more complete picture of the disclosure process and increase the applicability of our findings to real-world interactions.

### Overview of studies

In Study 1, we constructed a measure of self-reported online disclosure depth, and compared its associations to intimacy and satisfaction with those of self-reported offline disclosure depth. We also examined whether these associations between type of disclosure and relational outcomes differed according to the type of relationship (friend vs. romantic partner). In Study 2, we tested whether the associations between online disclosure depth and relational outcomes extended to the intimacy and satisfaction of the discloser’s partner. In other words, we looked into the association between the partner’s disclosure on the self. In Study 3, we sought to establish directionality and causality in the associations of online disclosure depth and relational outcomes by experimentally manipulating perceptions of online disclosure depth (low vs. high) by one’s partner. In Study 4, we tested one mechanism potentially underlying the association of online disclosure depth and relational outcomes by experimentally manipulating the *inclusivity of recipients* (low to high) of online disclosure. Finally, in Study 5 we identified one context that negated the effect of online disclosure depth on relational outcomes by experimentally manipulating the content (self-focused vs. partner/relationship-focused vs. friend-focused) of online disclosure.

## Study 1

In Study 1, we explored the association between online vs. offline disclosure and relational outcomes, intimacy and satisfaction, in the discloser. To do this, we used a pre-established self-report measure of offline disclosure and a self-report measure of online disclosure constructed for this study. For offline disclosure, we expected to replicate previous findings on disclosure’s positive effects on relationships [55–56], such that higher depth of disclosure would result in higher intimacy and satisfaction. As for online disclosure, we were unsure about the results, as previous findings are mixed [29, 42]. Because of this, we left our predictions regarding online disclosure open. We also investigated whether the association between type of disclosure and relational outcomes would depend on another contextual factor: The type of relationship, namely friend vs. romantic relationship.

Although different types of close relationships can share many characteristics, they are also known to have disparate rules, different characteristics, and generate differing expectations [57]. For example, romantic relationships are typically viewed in terms of passion and commitment, whereas friendships are largely viewed in terms of affiliation and fun [20, 58–60]. People expect more from their romantic partner than from their friends, in terms of exclusivity, discretion, commitment, and relationship maintenance behaviors [61–62]. Violations of relational expectations by a romantic partner are viewed much more negatively than violations by a friend [63–64]. Because of these differences in expectations and assumptions regarding romantic relationships and friendships, it is likely the type of relationship interacts with other contextual influences. Specifically, romantic relationships, being loaded with more meaning and expectations, would be more sensitive to the effects of other contextual factors. As such, we expected the connections between type of disclosure and relational outcomes will be stronger in romantic relationships as compared with friendships.

### Method

#### Participants

Sample size was determined using G*Power 3 software [65], which indicated at least 114 participants were needed to detect a small to medium-sized effect (*r* = .20-.30; based on previous findings of disclosure and relationship quality [66]) with 80% power and three predictors. One hundred and eighty-nine undergraduate students (66 men and 114 women, nine did not report gender), ranging in age from 18–31 (median = 19), participated in the study to earn course credit in their introductory psychology class. At the time, 106 were involved in a romantic relationship for over three months, and 83 were single. The University of Kansas Human Research Protection Program approved the study.

#### Materials and procedure

After consenting, participants completed a battery of self-report measures including measures assessing the depth of online disclosure, depth of offline disclosure, and relationship components (intimacy and satisfaction). For the offline disclosure and relationship components measures, participants completed them differently based on their romantic relationship status (described below). After completing these, participants provided demographic information and were asked to provide their consent to allow the researcher to view their Facebook page. They were then debriefed and thanked.

Constructed for the current study, the Online (Facebook and Twitter) Self-Disclosure Scale (S1 Appendix) is an eight-item measure that assesses the participant’s depth of online disclosure via Facebook and Twitter. Based on our definition of online disclosure as disclosure done in a public online space, the scale was created to capture the depth level of disclosure the participants put forth on their personal profiles/webpages on the online social network sites. Items included questions on how revealing and personal was the type of information participants tended to post, and participants rated the items on a 1 (*Not true of me at all*) to 7 (*Definitely true of me*) scale. The Cronbach alpha for the measure was adequate (α = .65) and hence one score of online self-disclosure was calculated by averaging the eight items. Higher scores represented higher depth of online disclosure.

We used a 10-item self-report measure [67] to assess the depth of offline disclosure, with the original rating scale modified to 1–7. Participants were asked to rate on a 1 (*do not discuss at all)* to 7 (*discuss fully and completely*) scale the amount to which they discuss certain topics with a close other. Examples of the topics included: deepest feelings, what they like and dislike about themselves, and things that are important to them in life. Higher ratings on these items reflect a higher depth level of offline disclosure. Participants completed the measure twice: Single participants completed the measure in regard to a same-sex and opposite-sex close friend, and coupled participants completed it in regard to a same-sex close friend and their romantic partner. Cronbach alphas for the offline disclosure scale were both high with regard to a male and a female close other (*α* = .93 and .93, respectively).

The Perceived Relationship Quality Components inventory (PRQC) [68] assesses levels of intimacy, commitment, trust, love, passion, and satisfaction in people’s close relationships. In the current study, we used only the intimacy and satisfaction subscales. Participants were asked to rate items such as “How intimate is your relationship?,” “How satisfied are you with your relationship?” on a scale ranging from 1 (*not at all*) to 7 (*extremely*). Higher ratings on these items reflect higher intimacy and satisfaction in the relationship. Participants were asked to complete this scale twice, once thinking about their romantic partner (if they did not have one they skipped this part), and once thinking about their best friend. Cronbach alphas for intimacy and satisfaction in friendship were .66 and .97, respectively; and .84 and .98 for intimacy and satisfaction with a romantic partner, respectively.

Finally, participants provided their demographics (gender, age, and relationship status) before moving on to a second informative consent form. The form explained to participants that the purpose of the current study was to better understand peoples’ use of social networking websites (e.g., Facebook) and how it may be associated with intimacy and satisfaction in close relationships. The form further detailed how the researchers were planning to investigate the amount of self-disclosure that individuals exhibit on their Facebook profiles and in an effort to not rely solely on self-report measures, the researchers would like to view the participants’ actual Facebook profiles (i.e., ‘walls’ at the time of study).

After assuring the participants that all of the information obtained from their profiles would be used strictly for research purposes and that the access they provided would be terminated following the study’s conclusion, they were asked to provide researcher access by way of befriending a temporary researcher account. Through this account, research assistants viewed and created screen snapshots of each profile’s first page. Using those, two independent raters then scored the extent to which each profile exhibited various aspects of disclosure using the items of the online self-disclosure measure (described previously). The reliability (intraclass correlation) between the two raters was high: .97, indicating a high level of inter-rater consistency. We then calculated an overall average score, which represented the objective rating of a participant’s online disclosure depth with higher values reflecting higher disclosure.

## Results and discussion

### Preliminary analysis: Objective ratings of online self-disclosure

We first sought to compare self-reported online disclosure and objective or other-evaluated disclosure. The correlation between the two scores was relatively high, *r*(46) = .51, *p* < .01, suggesting that the subjective scale we created is valid, and participants were both aware and open about their levels of self-disclosure. Because less than half of the people provided us with access to their Facebook page (a total of 48 pages were viable for analysis), we did not use the objective measure in other analyses. The correlation between online disclosure (via the self-report measure) and offline disclosure scores was *r*(181) = .13, *p* = .09. Although positive, the correlation was marginal and not very high, which suggests that online and offline disclosure are potentially working via different processes.

#### Main analysis

To examine the associations between disclosure and relational outcomes, we ran four regression analyses: two predicting intimacy (one within friendship and the other within romantic relationship) and two predicting satisfaction. We excluded six participants who were outliers on intimacy (defined as the residuals being over three standard deviations). In each regression, we controlled for gender (dummy coded as men = 1 vs. women = 2) based on the literature on gender differences in disclosure effects [69]. We included mean offline disclosure (computed by averaging the offline disclosure scores to a male and to a female close other) and online disclosure. See S1 Table for correlations between the variables.

The regression predicting intimacy with a romantic partner showed a main effect for online disclosure, *B* = -0.23, *t*(96) = -2.12, *p* = .037, such that the more one discloses online, the less intimacy he or she reports with his or her romantic partner. In addition, there was a main effect for offline disclosure, *B* = 0.31, *t*(96) = 3.23, *p* = .002, such that the more one tends to disclose offline, the greater is his or her reported intimacy with the partner.

The regression for satisfaction within one’s romantic relationships had a similar pattern of results. There was a main effect for online disclosure, *B* = -0.39, *t*(96) = -3.32, *p* = .001, such that the more one disclosed online, the lower was his or her satisfaction was in the romantic relationship. There was also a main effect for offline disclosure, *B* = 0.34, *t*(96) = 3.26, *p* = .002, such that the more one disclosed offline, the higher was his or her satisfaction with the partner.

Regarding friendships, the regression for the intimacy within a friendship revealed a main effect for gender, *B* = 0.44, *t*(170) = 2.22, *p* = .03, such that women reported greater intimacy in friendships. There was also a significant effect of offline disclosure, *B* = 0.35, *t*(170) = 4.33, *p* < .001, such that the more one disclosed offline, the greater intimacy he or she reported in his or her friendship. However, the regression did not reveal a main effect for online disclosure, *B* = -0.04, *t*(170) = -0.41, *p* = .68. Similarly, the regression for satisfaction in friendships revealed a significant effect for offline disclosure, *B* = 0.41, *t*(169) = 5.29, *p* < .001, showing that greater offline disclosure predicted higher satisfaction in friendships. However, again there was no main effect for online disclosure, *B* = -0.11, *t*(169) = -1.32, *p* = .19. In all of the regressions, no other effects were significant (Table 1).

**Table 1:** Study 1 regression analyses predicting relationship intimacy and satisfaction in romantic relationships and friendships.

Total Sample					Coupled Sample	
	Intimacy with Partner	Satisfaction with Partner	Intimacy with Friend	Satisfaction with Friend	Intimacy with Friend	Satisfaction with Friend
Predictor	*B*	*B*	*B*	*B*	*B*	*B*
**Gender**	0.06	0.25	0.44*	0.12	0.58*	0.21
**Offline self-disclosure**	0.31*^a^	0.34**	0.35***	0.41***	0.51***	0.51***
**Online self-disclosure**	-0.23*	-0.39**	-0.04	-0.11	-0.07	-0.10
***R***^***2***^	.13	.19	.16	.16	.23	.20
***N***	100	100	183	182	106	106

^a^ * *p* < .05, ** *p* < .01, *** *p* < .001.

As the romantic intimacy and satisfaction analyses were done only using coupled (vs. single) participants, whereas the friendship intimacy and satisfaction analyses were done with the entire sample, there was a possibility that discrepancies in factors determining romantic vs. friendship intimacy and satisfaction may have been due to participants’ relationship status rather than qualitative differences in friend vs. romantic relationships. To rule this out, we reran the regression analyses predicting friendship intimacy and satisfaction, using only participants who were in a romantic relationship. The results were similar to the ones we described above (Table 1), suggesting that as hypothesized the type of relationship rather than relationship status was accounting for the differences we found.

The results of Study 1 replicated past findings showing that greater offline disclosure predicts higher intimacy and satisfaction in close relationships. Unlike offline disclosure, online disclosure (operationalized as general profile disclosures) was negatively associated with intimacy and satisfaction, suggesting the influence of disclosure type on the association between disclosure and relational outcomes. Moreover, this negative association was found only in romantic relationships and not in friendships, providing evidence for contextual interaction effects. As our self-report measure of online disclosure was correlated with objective/observational ratings of online disclosure, these findings likely apply to actual online disclosure.

As disclosure is a dyadic process, whether the differential association of online disclosure and relational outcomes also extends to the partner is important to know to paint a whole picture of disclosure and relationships. Study 2 was designed to test this question.

## Study 2

Disclosure has been theorized to affect not only the relational outcomes of the discloser [19] but also the partner’s [18]. This has been supported in studies using offline disclosure in romantic [21, 55] and friend [70] relationships. These theoretical models and findings highlight the importance of the interplay between the partners, and as such in Study 2 we examined how online disclosure associates with the intimacy and satisfaction of the discloser’s romantic partner.

Based on the negative associations between online disclosure and the discloser’s intimacy and satisfaction in Study 1, and previous studies finding negative associations between the romantic partner’s perceived online social network use and one’s intimacy and satisfaction [42], we predicted that higher online disclosure would be negatively associated with the partner’s intimacy and satisfaction. We also reached out to both partners rather than basing the assessments on one party (a limitation of Hand et al. [42], who asked participants to report on their partner’s perceived online social network usage). As such, we were able to test the association between self-reported online disclosure (rather than the amount perceived by the partner) and the partner’s intimacy and satisfaction. It might be the case that reported online disclosure is actually less accurate than a partner’s report, as people can be biased about themselves as well. However, the results of Study 1 suggest that our measure is doing a relatively accurate job assessing disclosure, as it is correlated (*r*(46) = .51, *p* < .01) with objective ratings of neutral raters.

Because we measured the intimacy and satisfaction of the participants’ romantic partners, we decided to control for participants’ attachment styles. Studies have suggested that relational personality traits, such as attachment style, play a central role in the tendency to disclose, the amount of information being disclosed, and reactions to disclosure by others [71–75]. Importantly, attachment style has been associated with disclosure of the partner [71]. Specifically, the attachment anxiety of a participant was negatively associated with the romantic partner’s breadth and depth of disclosure during a seven day period. For a more stringent test of the effects that participants’ tendency to disclose online had on their partner’s intimacy and satisfaction, we aimed to control for the contribution of attachment style and examine whether our effects would still hold.

### Method

#### Participants

Sample size was determined using G*Power 3 software [65], which indicated at least 45 participants were needed to detect a large-sized effect (*r* = .50; based on previous findings of disclosure partner effects and relationship quality [21]) with 80% power and five predictors. Seventy-two undergraduate students (22 men and 50 women) ranging in age from 18–25 (median = 19) participated in the study to earn course credit in their introductory psychology class. All were involved in a romantic relationship for more than three months (*M* = 16.87, *SD* = 12.65, range 3–55; S1 Text). The University of Kansas Human Research Protection Program approved the study. All participants consented to allow the experimenter to contact their partner and get him/her to complete the PRQC [68] intimacy and satisfaction subscales (in return for an additional credit).

#### Materials and procedure

After consenting, participants completed a battery similar to the one we used in Study 1, including measures assessing online and offline disclosure, and an adult attachment scale. After completing these measures, participants provided demographics and were asked to provide their partner’s contact information. Partners, contacted via e-mail, were asked to complete the intimacy and satisfaction subscales of the PRQC [68]. Cronbach alphas for all scales in the current study were adequate to high: for the online disclosure, alpha was .72; for offline (male and female) disclosure tendencies, they were .91 and .92, respectively; for intimacy and satisfaction of partner they were .75 and .97, respectively.

The Experiences in Close Relationships scale (ECR) [76], a 36-item self-report measure of adult attachment, was used to evaluate participants’ attachment anxiety and avoidance (18 items in each subscale). Participants were asked to rate using a 1 (*Strongly disagree*) to 7 (*Strongly agree*) scale their agreement with each of the items regarding their close relationships. For example, the item “I worry that others won’t care about me as much as we care about them” reflects attachment anxiety, whereas “Just when someone starts getting close to me, I find myself pulling away” represents attachment avoidance. Higher scores on each scale reflect higher attachment insecurity, whereas lower scores on both scales reflect attachment security. In the current study, alphas for both scales were high (.93 and .91 for anxiety and avoidance; the two scores were moderately correlated *r*(70) = .35, *p* = .001).

#### Results and discussion

To test the associations between online disclosure and partner’s relational outcomes, we ran two regression analyses: one predicting the partner’s intimacy and the other predicting the partner’s satisfaction. In each regression, we entered gender (dummy coded as men = 1 vs. women = 2), attachment avoidance and anxiety, offline disclosure (computed again by averaging self-disclosure scores to a male and a female close other), and online disclosure. We excluded six participants who were outliers on intimacy (again having residuals being over three standard deviations). See S2 Table for correlations between the variables.

The regression predicting the partner’s intimacy revealed a main effect for online disclosure, *B* = -0.28, *t*(58) = -2.96, *p* = .004, such that the more one discloses online, the less intimacy his or her partner reported. In addition, there was a main effect for attachment anxiety, *B* = 0.20, *t*(58) = 2.60, *p* = .01, such that the more anxiously attached someone was, the higher intimacy his or her partner reported. There was also an effect for attachment avoidance, *B* = -0.37, *t*(58) = -3.76, *p* < .001, such that the more avoidant a person was, the less intimacy his or her partner reported.

The regression predicting the partner’s satisfaction also showed a significant main effect for online disclosure, *B* = -0.21, *t*(58) = -2.04, *p* = .046, such that the more one disclosed online, the lower his or her partner’s satisfaction was. No other effects were significant in both analyses (Table 2).

**Table 2:** Study 2 regression analyses predicting romantic partner’s relationship intimacy and satisfaction.

	Partner’s Intimacy	Partner’s Satisfaction
Predictor	*B*	*B*
**Gender**	-0.16	-0.20
**Attachment avoidance**	-0.37***^a^	-0.19
**Attachment anxiety**	0.20*	0.04
**Offline self-disclosure**	0.14	0.20
**Online self-disclosure**	-0.28**	-0.21*
***R***^***2***^	.31	.15
***N***	66	66

^a^ * *p* < .05. ** *p* < .01. *** *p* < .001.

We also found that attachment anxiety was positively related to intimacy, and avoidance was negatively related to intimacy. These results are in line with previous literature that has documented mixed associations of anxiety with intimacy [71, 77–79] and negative associations of avoidance with intimacy [77].

As predicted, in Study 2 we found that higher online disclosure (operationalized as general profile disclosures) negatively associated with the partner’s intimacy and satisfaction. Together, the findings of Studies 1 and 2 show the influence of contextual factors on the association between disclosure and relational outcomes reaches both the discloser and his or her partner.

While uncovering the nature of the associations between disclosure type and relational outcomes, the findings of Studies 1 and 2 do not shed light on the direction of those associations nor the causal relationship. In Study 3 we conducted an experiment to assess if perceiving one’s partner to highly disclose online would indeed lower intimacy and satisfaction in the relationship.

## Study 3

To test whether perceived partner online disclosure affected the recipient’s intimacy and satisfaction, in Study 3 we used an experimental approach—manipulated the depth of disclosure one’s partner supposedly engaged in online—and subsequently measured participants’ intimacy and satisfaction. We constructed mock Facebook pages (‘walls’ or ‘timelines’) representing either a high or low depth level of the owner’s disclosure. In line with Collins and Miller’s [43] review on experimental manipulations of disclosure depth, a high depth level of disclosure was operationalized by including more personal and emotional information on the page; whereas low depth disclosure was operationalized by including less personal and emotional information (see details below). We conducted a pretest of the primes in order to make sure they differed on perceived disclosure depth, and not on other dimensions such as appeal of the page, negative mood, or inappropriateness. This was important because public high depth disclosure can be seen as being inappropriate [80]. To test this, we included three questions about the appropriateness of the messages in the pretest.

We next exposed participants in romantic relationships to one of these primes (high vs. low disclosure) and asked them to imagine that the Facebook page belonged to their partner. After this priming procedure, we measured participants’ relationship intimacy and satisfaction. We predicted that exposure to the Facebook page inducing higher perceived partner online disclosure would result in lower intimacy and satisfaction, as compared with exposure to the low disclosure Facebook page.

### Method

#### Participants

Sample size was determined using G*Power 3 software [65], which indicated at least 77 participants were needed to detect a medium-sized effect (*r* = .30; based on previous findings of perceived partner disclosure and relationship quality [66]) with 80% power and three predictors. 132 adults (55 men and 77 women), ages 18 to 69 (median = 22), participated in the study on a voluntary basis, recruited using a snowballing procedure. All participants were involved in a romantic relationship for more than three months (*M* = 52.09, *SD* = 84.68, range 3–564) at the time of the study. The University of Kansas Human Research Protection Program approved the study.

#### Materials and procedure

Participants were exposed to one of two mock Facebook pages (see details below). They then completed a battery of self-report measures similar to the one we used in Studies 1 and 2, including measures assessing intimacy and satisfaction in close relationships (modified to fit the instructions of the prime), offline disclosure (however, we reverted to using the original rating scale from 0 (*do not discuss at all)* to 4 (*discuss fully and completely*)), and demographics. At the end of the study, they were given a debriefing form and thanked. Cronbach alphas for all scales in the current study were adequate to high, reflecting high reliability: for intimacy and satisfaction alphas were .94 and .92, respectively, and for offline disclosure the alpha was .90.

Using actual Facebook page screenshots and a photo-editing program, we constructed two versions of mock Facebook ‘wall’ pages (see https://osf.io/kvndp/ for a copy of the stimuli). The pages were made so they were similar in all components, including information breadth, with the exception of the depth of disclosure. Breadth was operationalized via the number of posts, and depth via the topics. The owner of the ‘wall’ had a gender-neutral name (“Alex”), was of college age (birth year 1991, age 21 at time of study as the majority of our participants were expected to be students), and had posted six to nine updates. In the high disclosure condition, the ‘wall’ was comprised of pictures of people posing for the camera during a party (which were taken from the Internet) and status updates containing personal information (e.g., “Getting close to my goal weight!,” “So we had a pretty bad fight with Mom,” “Had a really nice night today, you guys are awesome!" “3 hours of training at work, pretty interesting,” “This psyc class is frustrating… I’m just mad that’s all”). In the low disclosure condition, the ‘wall’ was made of pictures of puzzles, news article links (likewise taken from the Internet) and status updates containing relatively impersonal information (e.g., “Perfect weather today,” “I love beer”). Both ‘walls’ were constructed with materials from actual Facebook pages. In a pretest, nine participants (5 males, ages 18–21) expressed the ‘walls’ seemed to be actual pages of real people.

In a second between-subjects pretest of the primes, 31 participants (3 men and 28 women, median age = 24, range 20–45) answered 15 questions (e.g., “How much do you think this person is self-disclosing?”) regarding the walls, using a 7-point Likert scale, ranging from 1 (*Not at all*) to 7 (*Very much*). We found no significant differences between the ‘walls'‘ various aspects, such as liking of the ‘wall’ owner, obnoxiousness of owner, and amount of information the ‘wall’ contained (controlled for equal breadth of disclosure). However, as intended, the ‘walls’ differed on how much the owner was perceived to be self-disclosing, *t*(29) = 2.73, *p* = .01, Cohen’s *d* = -1.01, in that the owner of the high disclosure ‘wall’ was rated as disclosing more (*M* = 4.72, *SD* = 1.36) than the low disclosure ‘wall’ (*M* = 3.46, *SD* = 1.13) (see S4 Table and S2 Text).

In the actual study, participants were presented with one of the mock ‘wall’ pages using a computer screen and received the following instructions: “Try to imagine that the following Facebook wall is your partner’s. During the last few weeks, your partner has been constantly posting personal information like that shown in the example below.” The word “personal” was only included in the high disclosure condition. This was in order to increase the strength of the manipulation.

### Results and discussion

To test whether perceived partner online disclosure on Facebook predicted relationship intimacy and satisfaction, we ran two regression analyses: one predicting intimacy, and the other predicting satisfaction. We entered gender (dummy coded as men = 1 vs. women = 2), offline disclosure, and disclosure prime (dummy coded as low = 0 vs. high disclosure = 1). Five participants were excluded from the analysis due to being outliers on intimacy (having residuals greater than three standard deviations). See S3 Table for correlations between the variables.

**Fig 1:**
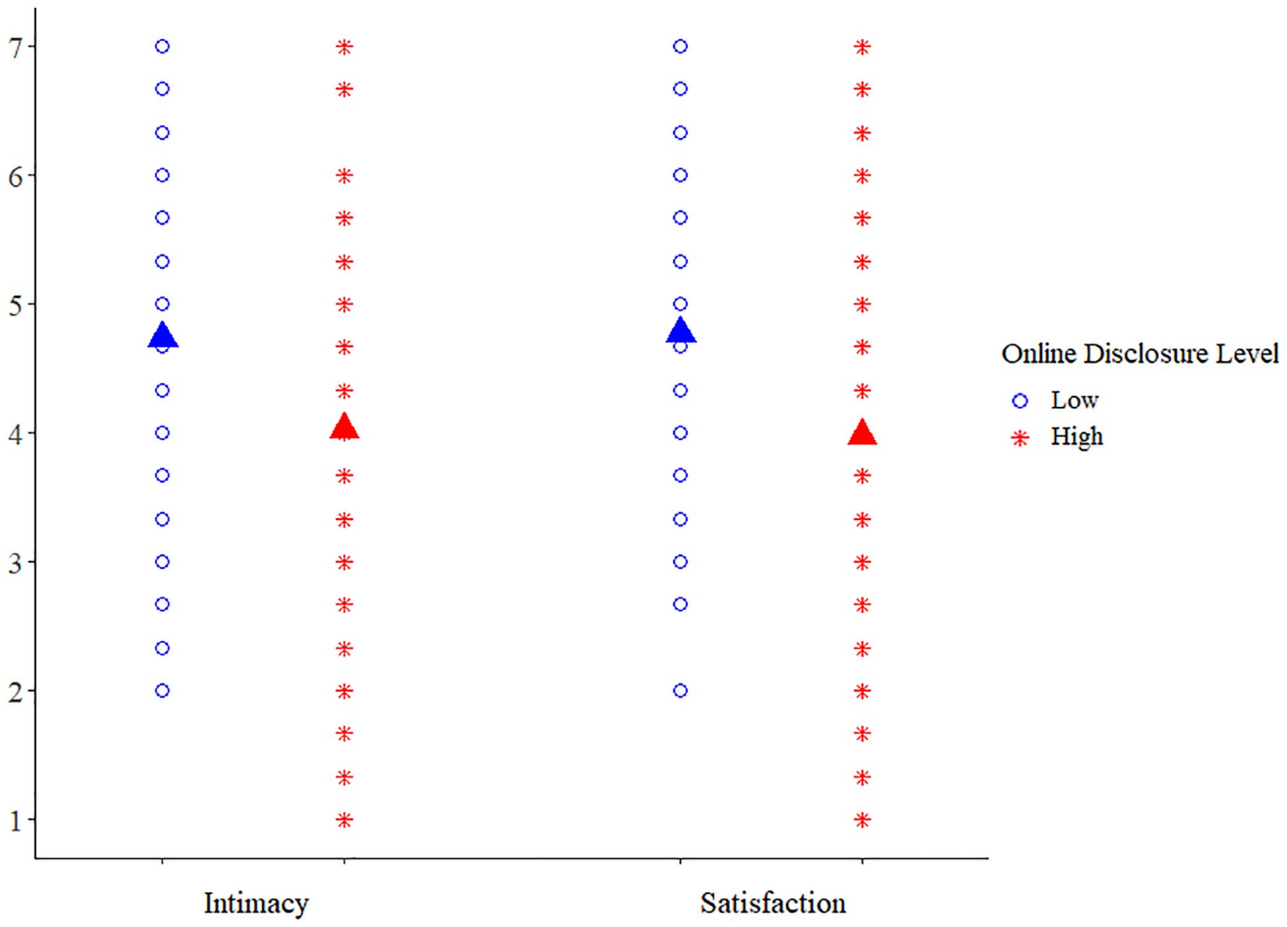
Study 3 means for intimacy and satisfaction in each of the prime conditions.

The regression predicting intimacy revealed a main effect for prime, *B* = -.70, *t*(123) = -2.55, *p* = .01, such that those exposed to the high disclosure prime reported lower intimacy (*N* = 62, *M* = 4.03, *SD* = 1.64) compared to those exposed to the low disclosure prime (*N* = 65, M = 4.57, *SD* = 1.55). The prime also significantly predicted differences in satisfaction, *B* = -.79, *t*(123) = -3.04, *p* = .003, such that those exposed to the high disclosure prime reported lower satisfaction (*M* = 3.98, *SD* = 1.57), compared to participants exposed to the low disclosure prime (*M* = 4.57, *SD* = 1.53) (see Fig 1 for scatterplot and means according to disclosure depth level for intimacy and satisfaction). No other effects were significant (Table 3).

**Table 3:** Study 3 regression analyses predicting relationship intimacy and satisfaction.

	Intimacy	Satisfaction
Predictor	*B*	*B*
**Gender**	0.04	0.02
**Offline self-disclosure**	-0.02	0.06
**Disclosure prime**	-0.70*^a^	-0.79**
***R***^***2***^	.05	.07
***N***	127	127

^a^ * *p* < .05. ** *p* < .01. *** *p* < .001.

In Study 3, using an experimental design, we found support for the proposition that being exposed to the information that one’s partner highly discloses online (in the form of timeline photos and status updates) would lower intimacy and satisfaction in individuals’ romantic relationships.

Studies 1–3 have demonstrated the interactional effects of contexts on the association between disclosure and relational outcomes. As mentioned earlier, to resolve inconsistencies in the literature on online disclosure and relationships, and to better predict contextual effects of disclosure, it is important to identify underlying mechanisms that drive them. In Study 4, we examined a potential factor that might account for the negative effects of online disclosure on intimacy and satisfaction: the inclusivity of recipients.

## Study 4

A key structural feature of online disclosure is its high inclusivity [11–13]. *Inclusivity* refers to the extent to which the disclosed information is available vs. restricted to recipients. A disclosure made to one recipient is low in inclusivity, whereas a disclosure made to many people is high in inclusivity. The idea of inclusivity is similar to constructs such as *media reach*—the size of a medium’s attainable audience [3]—and *imagined audience*—those for whom the message is constructed [81]. It is also similar to *personalism*—the concept of a message or behavior being specifically directed toward an individual(s) [82]. Inclusivity, however, is different from these other constructs. For example, it differs from *media reach* in that it refers to the current number of recipients, rather than the maximum possible via the medium. It also differs from *imagined audience*, which is decided and perceived by the discloser. Inclusivity is also decided by the discloser, but importantly for the current paper, it can and often is perceived by both the discloser and the recipient. Inclusivity also differs from *personalism* in that it does not involve any assumptions regarding how much the message was tailored for the self, and whether or not the self was specially chosen for the disclosure, whereas personalism does.

As previously mentioned, online disclosure has a high level of inclusivity compared to offline disclosure [12–13]. For example, Facebook and Twitter tend to have numerous recipients, with an average of over 300 people for a typical disclosure in each service, and a maximum at several thousands or millions, respectively [83–84]. This higher inclusivity can affect the influence of the disclosure on relational outcomes by changing the overall interpretation of the content. For example, it may make high-depth disclosure from a romantic partner seem less special. Violations of expectations regarding romantic relationships are associated with strong negative feelings [63–64], which may decrease intimacy, and in turn, satisfaction [85].

In line with this research, Bazarova [80] manipulated the inclusivity of disclosure and found that observers judged the discloser and recipient to possess lower friendship intimacy when the disclosed message was having many recipients as opposed to having one recipient. However, Bazarova did not test whether this finding can be applied to romantic relationships. In addition, assuming the role of an observer is one step removed from imagining yourself in the actual situation, so a study measuring actual intimacy in the recipient will likely yield stronger and more valid effects. More importantly, Bazarova did not test whether perceptions of disclosure inclusivity accounted for the difference in intimacy ratings, so it is difficult to know what process underlies her findings.

Perceptions of low inclusivity in disclosure are likely to positively affect relational outcomes, whereas perceiving disclosure as high in inclusivity is likely to result in negative relational outcomes. As the type of online disclosure we are interested in here is usually done in front of a broader audience, participants will likely perceive their partner’s disclosure as being high on inclusivity. We predicted that this would result in lower intimacy and in turn lower satisfaction in the relationship, as compared with disclosure that is low in inclusivity.

### Method

#### Participants

Sample size was determined using G*Power 3 software [65], which indicated at least 123 participants were needed to detect a medium-sized effect (r = .30; based on previous findings of perceived partner disclosure and relationship quality [66] and Study 3 effect sizes for online disclosure (Cohen’s *d* = 0.46–0.55) with 80% power and 11 predictors. 143 adults (76 men and 66 women, 1 unreported, median age = 30, range 19–70) took part in the study, for compensation via Amazon mTurk. Four people were excluded because they were not in an exclusive relationship of at least three months. All others were presently involved in a romantic relationship which had lasted more than three months (*M* = 73.46 months, *SD* = 74.38, range 3–417). The University of Kansas Human Research Protection Program approved the study.

#### Materials and procedure

Participants were exposed to one of ten mock Facebook messages and instructions (see details below). Next, they answered a question regarding how inclusive/exclusive they thought the message was (inclusivity question) using a 7-point Likert scale ranging from 1 (*Not at all exclusive*) to 7 (*Very exclusive*). Participants then completed the intimacy (*α* = .93) and satisfaction (*α* = .95) items of the PRQC and the offline self-disclosure measure (*α* = .89). Finally, participants answered demographic questions and were debriefed and thanked.

We created five different pairs of mock Facebook messages to use in the primes. The posts were made to vary only in the depth of disclosure (high/low as in Studies 1–3) and in the inclusivity of the disclosure. To manipulate inclusivity, we altered the instructions associated with the primes. There were five levels of inclusivity, ranging from low to high. The lowest inclusivity primes instructed participants to imagine that their partner had sent the messages only to them; second lowest—to them and one other recipient; medium inclusivity—to them and 4 others; second highest—to them and 19 others; and the highest inclusivity primes—to them and 24 others (how these numbers were determined is explained below). To manipulate the depth of disclosure, we altered the message content of the primes. High disclosure primes contained more emotional content and personal information (e.g., “Had a real nice night tonight! You guys are awesome.”) relative to the low disclosure primes, which were more impersonal (e.g., “Let’s go team! Great win tonight.”). Therefore, there were ten different primes (see S2 Appendix), resulting in a 2 (levels of disclosure) x 5 (levels of inclusivity) study design.

The number of recipients assigned to each condition were based on a pretest of 53 participants (32 men and 21 women, median age = 19, range 18–28), set to determine the numbers of recipients that would produce different perceptions of inclusivity. Participants of the pretest were randomly presented with 4 of 12 primes that contained mock Facebook messages that varied only in the number of recipients from 1 to 250 (1, 2, 3, 4, 5, 10, 20, 25, 50, 75, 100, 150, and 250). The highest number, 250, was decided on because in the year of the study (2014) this was the maximum number of recipients Facebook allowed a group message to be sent to. We started from 1 recipient (dyad) to 2 (triad; based on Solano & Dunnam [86]), and then increased the numbers until 250. For each of the primes, participants were asked to rate how exclusive they thought the message was, on a 7-point scale ranging from 1 (*Not exclusive at all*) to 7 (*Very exclusive*). We examined the data using a scatterplot to determine the cutoff numbers in which the pretest scores became similar. The scatterplot showed the scores differed between 1 and 2, 2 and 5, 5 and 20, 20 and 25, and levelled off after 25. As such, we selected the following numbers that affected perceived inclusivity: 1, 2, 5, 20, and 25.

In a second between-subjects pretest, 20 participants (9 men and 11 women, median age = 30, range 19–60) were asked to evaluate six aspects of the high vs. low disclosure messages. As expected, those exposed to the high disclosure messages perceived the sender as more disclosing (*M* = 4.80, *SD* = 1.14) relative to those exposed to the low disclosure messages (*M* = 2.90, *SD* = 1.60), *t*(18) = -3.07, *p* = .007, Cohen’s *d* = -1.45 (see S6 Table for full array of questions).

### Results and discussion

To see if our inclusivity prime changed perceptions of inclusivity (manipulation check), we conducted a one-way analysis of variance (ANOVA) with inclusivity prime as a predictor. For the dependent variable we used the inclusivity question, with answers recoded so that high scores reflected high perceptions of inclusivity. See S5 Table for correlations between the variables. Results showed a main effect for prime on perceived inclusivity, *F*(4, 127) = 4.78, *p* = .002. Post-hoc analysis using Tukey’s HSD test showed the lowest inclusivity prime (1 recipient; *M* = 4.14, *SD* = 1.85) significantly differed from the 20 recipient prime (*M* = 5.93, *SD* = 1.46), *p* = .001, and marginally different from the 25 recipient prime (*M* = 5.43, *SD* = 1.90), *p* = .05, in that inclusivity was lower in the 1 recipient compared to the 20 and 25 recipient primes (see Table 4 for means and SDs of all inclusivity conditions).

**Table 4:** Study 4 means and standard deviations of intimacy, satisfaction, and perceived inclusivity of messages according to number of recipients.

		1 recipient (*N* = 29)	2 recipients (*N* = 23)	5 recipients (*N* = 27)	20 recipients (*N* = 30)	25 recipients (*N* = 24)
**Intimacy**	**Low disclosure**	5.07 (1.79)	5.33 (1.52)	5.71 (1.51)	5.64 (1.66)	4.86 (1.67)
	**High disclosure**	5.57 (1.22)	5.12 (1.54)	5.26 (1.66)	5.56 (1.17)	4.03 (1.66)
**Satisfaction**	**Low disclosure**	4.96 (1.85)	5.36 (1.35)	5.83 (1.41)	5.64 (1.88)	5.00 (1.48)
	**High disclosure**	5.79 (1.52)	5.15 (1.31)	5.56 (1.51)	5.16 (1.55)	4.12 (1.47)
**Perceived inclusivity**		4.14 (1.85)	4.82 (1.40)	4.70 (1.81)	5.93 (1.46)	5.42 (1.86)

To test the hypothesis that inclusivity accounts for the negative effects of high online disclosure on intimacy and satisfaction, we ran two hierarchical regression analyses, the first one predicting intimacy and the second one predicting satisfaction. We excluded six participants who were outliers on intimacy, defined as the residuals being more than three standard deviations. In the first step of each regression, we entered gender (dummy coded as men = 1 vs. women = 2), offline disclosure, online disclosure level of prime (coded as low = 0 vs. high = 1), and inclusivity level of prime (dummy coded into four separate variables with the lowest inclusivity level as the baseline). In the second step, we entered the interaction terms of disclosure level and each of the dummy codes for the four higher inclusivity levels.

The regression analysis predicting intimacy with a romantic partner revealed a main effect of the highest (25 recipients) inclusivity level prime, *B* = -0.86, *t*(124) = -2.21, *p* = .03, such that participants who were exposed to the highest (25 recipients) inclusivity level prime reported lower intimacy (*M* = 4.46, *SD* = 1.68) than did participants exposed to the lowest (1 recipient) inclusivity prime (*M* = 5.31, *SD* = 1.54). This effect was qualified by an interaction with online disclosure level (high/low), *B* = -1.58, *t*(120) = -2.02, *p* = .046. Probing the interaction using Preacher, Curran, and Bauer’s [87] web computational tool found that the effect of the highest inclusivity prime was only significant in the high disclosure condition, *B* = -1.67, *t*(120) = -3.00, *p* = .003. In addition, offline disclosure positively predicted intimacy, *B* = 0.87, *t*(120) = 5.45, *p* < .001, such that the greater the level of offline disclosure, the more intimacy the participant reported. No other effects were significant.

**Fig 2:**
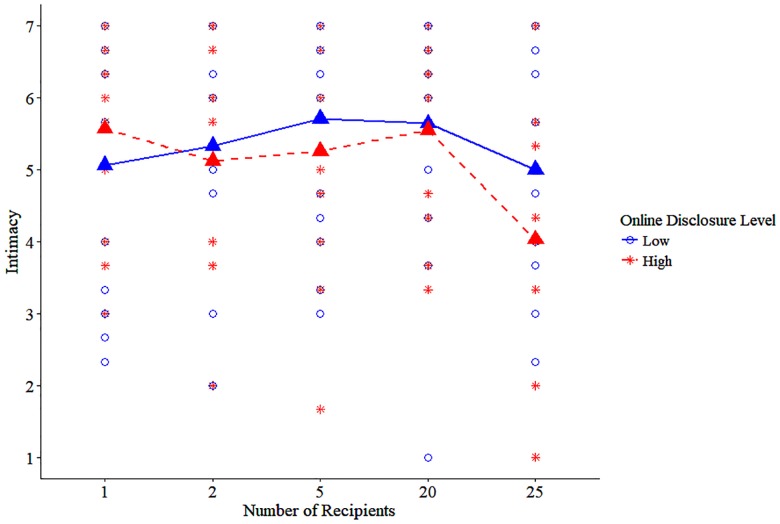
Study 4 means for intimacy in each of the prime conditions.

**Fig 3:**
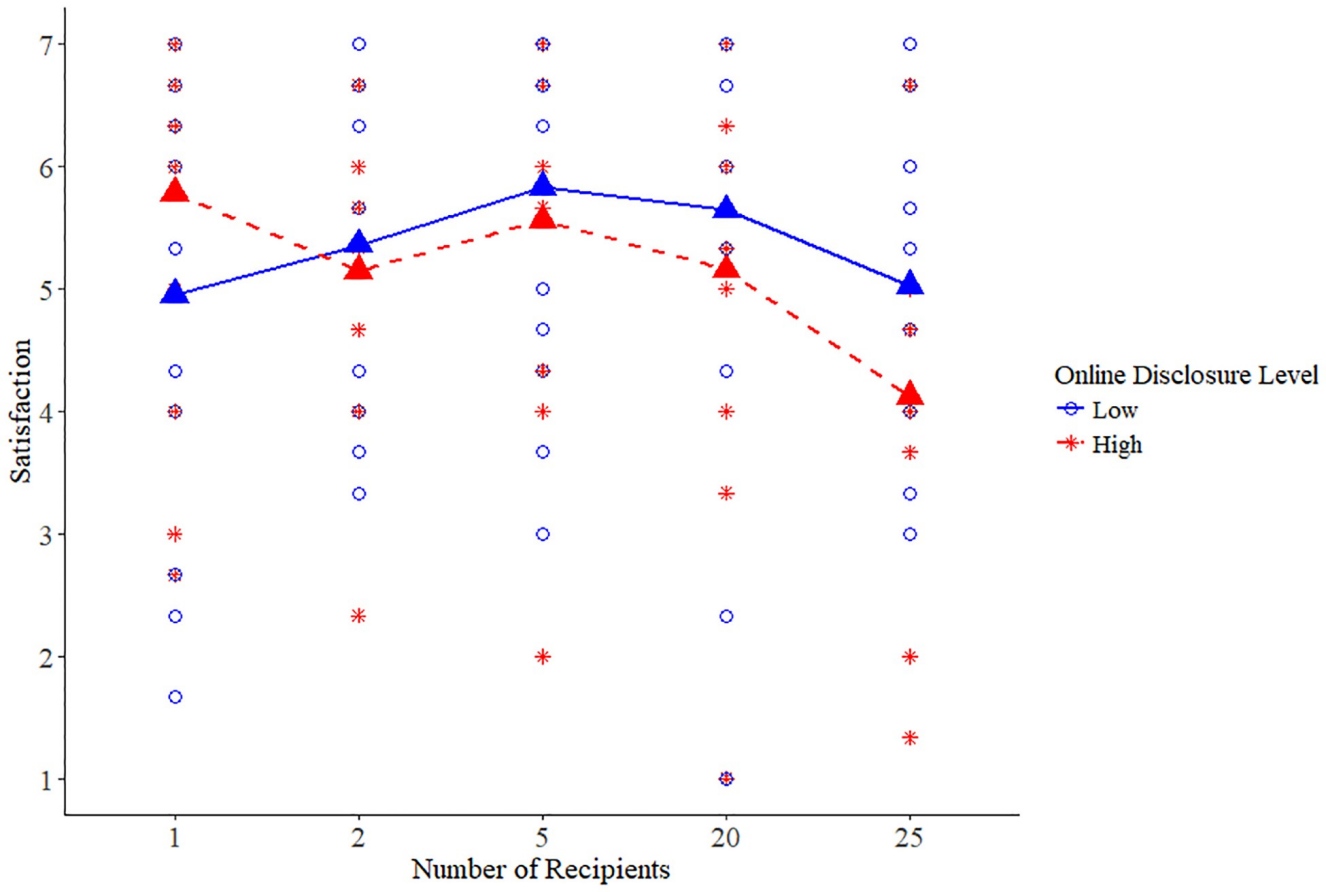
Study 4 means for satisfaction in each of the prime conditions.

The regression analysis for satisfaction revealed similar results, with a main effect of the highest (25 recipients) inclusivity level, *B* = -0.83, *t*(124) = -2.11, *p* = .04, such that participants who saw the highest inclusivity instructions reported lower satisfaction (*M* = 4.57, *SD* = 1.51) than did participants exposed to the lowest inclusivity prime (*M* = 5.36, *SD* = 1.72). This effect was also qualified by an interaction with online disclosure level, *B* = -1.90, *t*(120) = -2.43, *p* = .02. Again, probing the interaction using the same web calculator [87] showed the effect of highest inclusivity level on satisfaction was only significant in the high disclosure condition, *B* = -1.81, *t*(120) = -3.24, *p* = .002. The interaction term of the 20 recipient prime and disclosure was also significant, *B* = -1.62, *t*(120) = -2.23, *p* = .03, suggesting that these effects of inclusivity may start as early as 20 recipients are included (see Figs 2 and 3 for scatterplot and means according to inclusivity condition for intimacy and satisfaction, respectively).

In addition, offline disclosure positively predicted satisfaction, such that higher levels of offline disclosure were associated with higher reported satisfaction, *B* = 0.93, *t*(120) = 5.85, *p* < .001. No other effects were significant (see Table 5). Our results show that a high (over 25 for intimacy, over 20 for satisfaction) inclusivity of recipients is required for the negative effects of high-depth online disclosure on relationship intimacy and satisfaction to occur. In other words, the inclusivity of recipients is a mechanism that underlies the detrimental effects of high-depth online disclosure.

**Table 5:** Study 4 hierarchical regression analyses predicting relationship intimacy and satisfaction.

	Intimacy		Satisfaction	
Predictor	*ΔR*^*2*^	*B*	*ΔR*^*2*^	*B*
**Step 1**	.25***^a^		.25***	
**Gender**		0.11		-0.11
**Offline self-disclosure**		0.86***		0.90***
**Online disclosure level**		-0.16		-0.14
**2 recipient prime**		0.17		0.20
**5 recipient prime**		0.41		0.57
**20 recipient prime**		0.36		0.10
**25 recipient prime**		-0.86*		-0.83*
**Step 2**	.03		.04	
**Gender**		0.17		-0.06
**Offline self-disclosure**		0.87***		0.93***
**Online disclosure level**		0.66		0.93
**2 recipient prime**		0.51		0.63
**5 recipient prime**		0.91		1.08*
**20 recipient prime**		0.82		0.89
**25 recipient prime**		-0.10		0.09
2 recipient prime*Disclosure		-0.72		-0.89
5 recipient prime*Disclosure		-1.03		-1.05
20 recipient prime*Disclosure		-0.93		-1.62*
25 recipient prime*Disclosure		-1.58*		-1.90*
**Total *R***^***2***^	.28		.29	
***N***	133		133	

^a^ * *p* < .05. ** *p* < .01. *** *p* < .001.

In Study 4 we found that as predicted, perceptions of a partner’s high depth online disclosure (operationalized as private messages) led to lower intimacy and satisfaction in the receiver, but only when the disclosure was high in inclusivity. There was no main effect of online disclosure level, which was expected because the findings in Study 3 of high disclosure leading to lower intimacy and satisfaction were contingent on the messages being highly inclusive. This suggests that the high number of recipients involved in online disclosure accounts for its differential effects (compared to offline disclosure) on relational outcomes, rather than the disclosure being done through an electronic medium.

In Study 4, we successfully identified one mechanism behind the effects of online disclosure: the inclusivity of recipients. Uncovering this mechanism allows us to understand why online disclosure is having a negative effect on relationships and also enables the prediction of contextual outcomes. For example, although it would be a rarer situation, we can expect high depth offline disclosure done in the face of many recipients to also have negative effects on relationships.

Together, Studies 1–4 suggest that disclosing highly online will likely hurt one’s romantic relationship. However, this does not explain the findings on positive consequences of online disclosure in relationships [29–30, 36]. Those findings could be explained by another contextual factor that ameliorates or negates the negative effects of online disclosure on relationships: content focusing on the relationship or partner. In our studies, the content of disclosure was mainly focused on the self. For example, the high depth disclosure included in the primes used in Studies 3–4 did not include any information about the discloser’s romantic relationship or the partner. As such, in Study 5 we used an experimental design to investigate whether the focus of the disclosure content could affect the association between high online disclosure and relational outcomes.

## Study 5

One way to categorize the content of disclosure is to distinguish between information that focuses solely on the self (*personal disclosure*) and information regarding relationships/interactions with others (*relational disclosure*) [31, 88–89]. Although not much research has been done on this topic, a few studies suggest that relational disclosure, compared to personal disclosure, can bring positive consequences for relationships. For example, displaying pictures taken with one’s partner or disclosing the romantic relationship status on online social networks was associated with higher relationship satisfaction for both the discloser and the partner [29–30, 36]. On the other hand, viewing self-focused online disclosure can make one’s partner feel left out of the discloser’s life. This feeling of exclusion [90] may be detrimental to relationships. Supporting this idea, one study found when participants imagined that their romantic partners did not have any couple photos on Facebook, feelings of anger increased [91]. This suggests that the content of personal information disclosed would matter for the effect of perceived online disclosure on relationship quality. In other words, any negative effect of online disclosure may be canceled out by positive relational content. In Study 5 we investigated this possibility by manipulating the focus of the information disclosed online.

We further wanted to examine whether disclosing about one’s partner or the romantic relationship has unique positive effects, or whether disclosing about anyone else or any relationship (e.g., a friendship) would result with similar positive outcomes. To test this, we constructed two new mock Facebook ‘walls’ with status updates that had highly disclosing information about one’s romantic relationship/partner (high disclosure partner-focused prime) or one’s friendships/friends (high disclosure friend-focused prime). The effects of the partner-focused and friend-focused primes on relationship intimacy and satisfaction were compared to that of the self-focused disclosure prime (the high disclosure prime used in Study 3). We focused only on comparing high disclosure conditions in Study 5 because Studies 3 and 4 showed that the negative effects of disclosure on relationship outcomes happened mainly in the high disclosure conditions. We hypothesized that compared to the self-focused prime, the partner-focused prime will increase romantic intimacy and satisfaction, whereas the friend-focused prime will show no difference with the self-focused prime. This is because we presumed that the negative effect of online disclosure on the romantic relationship is not due to the fact that the discloser is self-focused, but rather that the discloser is excluding disclosure about the romantic partner.

### Method

#### Participants

Sample size was determined using G*Power 3 software [65], which indicated at least 104 participants were needed to detect a medium-sized effect (r = .30) with 80% power, three groups, and two covariates. 156 adults (59 men and 97 women), ages ranging from 18 to 61 (median = 20) participated in this study. The participants were either recruited from Amazon mTurk (*N* = 66) or undergraduate students participating for class credit (*N* = 90). All were involved in a romantic relationship for over three months, ranging from three to 243 months (*M* = 31.22, *SD* = 37.87). The University of Kansas Human Research Protection Program approved the study. We excluded one participant because the person did not complete the dependent measure.

#### Materials and procedure

Participants completed the study using the online survey software Qualtrics. They were randomly assigned to one of three conditions: a high disclosure self-focused prime (*N* = 53), a high disclosure partner-focused prime (*N* = 51), or a high disclosure friend-focused prime (*N* = 50; see details below). As in Study 3, participants were given one of the mock Facebook ‘wall’ pages with instructions asking them to study the ‘wall,’ while imagining their partner was its owner, and the message on it was either only about the partner, both partners, or the partner and friend(s). The instructions were identical to the ones used in the high disclosure condition of Study 3. Participants then completed the intimacy (α = .92) and satisfaction (α = .95) items of the PRQC and the offline self-disclosure measure (α = .91), as in previous studies. Finally, participants answered demographic questions, were debriefed and thanked.

Regarding the primes, the high disclosure self-focused prime was the same high disclosure prime used in Study 3. For the high disclosure partner-focused prime, the same format was used, but the status updates were changed to reveal information about the partner/relationship, such as “The weekend was good, my partner and I both enjoyed a nice boat ride!” and “Studying in the library today with my partner … this psych textbook is so boring …” In addition, the pictures in the ‘wall’ were changed to those of a couple. The same format was used for the high disclosure friend-focused prime, with the updates modified to display information about the owner’s friends, such as “The weekend was good, my friends and I enjoyed a nice boat ride!” and “Studying in the library today with my friends … this psych textbook is so boring …” For this prime, the pictures on the ‘wall’ were identical to the high disclosure self-focused prime’s as those pictures already included multiple people who could be construed as friends (see https://osf.io/kvndp/ for a copy of the stimuli).

A between-subjects pretest of the three primes was done with 32 participants (12 men and 20 women, median age = 24, range 19–56). The questions included those used in the prime pretest of Study 3. In addition, we added two questions asking the extent to which the romantic partner would feel “left out” after seeing the ‘wall,’ and the extent the romantic partner seems involved in the owner’s life. These questions were used in order to determine if the relational prime was indeed “partner-focused,” i.e., contained relatively more information about the romantic relationship and partner. We also included three questions regarding the appropriateness of the ‘wall’ content, as inappropriate disclosure has been shown to influence perceptions of the discloser [92–93]. The pretest results showed that the primes did not differ on any aspects, such as how much participants perceived the ‘wall’ owner to be disclosing, how personal the content of the ‘wall’ seemed to be, and appropriateness of the ‘wall’ content (see S8 Table for full array of questions). However, as we intended, the three ‘walls’ differed on how much the partner would feel “left out” after seeing the ‘wall’ (*F*(2, 29) = 4.53, *p* = .02), and how involved the romantic partner seemed to be in the owner’s life (*F*(2, 29) = 15.55, *p* < .001). Post-hoc analysis using Tukey’s HSD test found the high disclosure partner prime was perceived to make the partner feel relatively less “left out” (*M* = 3.45, *SD* = 1.44) than the high disclosure self prime (*M* = 1.82, *SD* = 1.08), *p* = .02, and the high disclosure friend prime (*M* = 1.82, *SD* = 1.08), *p* = .02. The romantic partner was seen to be more involved in the owner’s life in the partner-prime (*M* = 5.45, *SD* = 1.57) compared to the self- *(M* = 2.80, *SD* = 1.03) and friendship- (*M* = 2.36, *SD* = 1.50) primes, both *p’s* < .001.

### Results and discussion

To test whether self-focused vs. partner-focused vs. friend-focused high online disclosure had different effects on intimacy and satisfaction, we ran two analysis of covariance (ANCOVA) models, one predicting intimacy, and one predicting satisfaction. We included gender (dummy coded as men = 1 vs. women = 2), offline disclosure, and prime type as predictors. See S7 Table for correlations between the variables.

The ANCOVA predicting intimacy revealed a main effect for prime type, *F*(2, 149) = 10.28, *p* < .001. Main effects pairwise comparisons using Fisher’s LSD test showed significance differences between the partner-focused prime (*M* = 5.74, *SD* = 0.96) and self-focused prime (*M* = 4.5, *SD* = 1.63), *p* < .001, and friend-focused prime (*M* = 4.69, *SD* = 1.66), *p* < .001, such that being exposed to the partner-focused prime led participants to report higher intimacy compared to both the self-focused and friend-focused primes. In addition, offline disclosure positively predicted intimacy, such that the higher one’s tendency to disclose offline, the higher was one’s reported intimacy with the partner, *F*(1, 149) = 9.83, *p* = .002.

**Fig 4:**
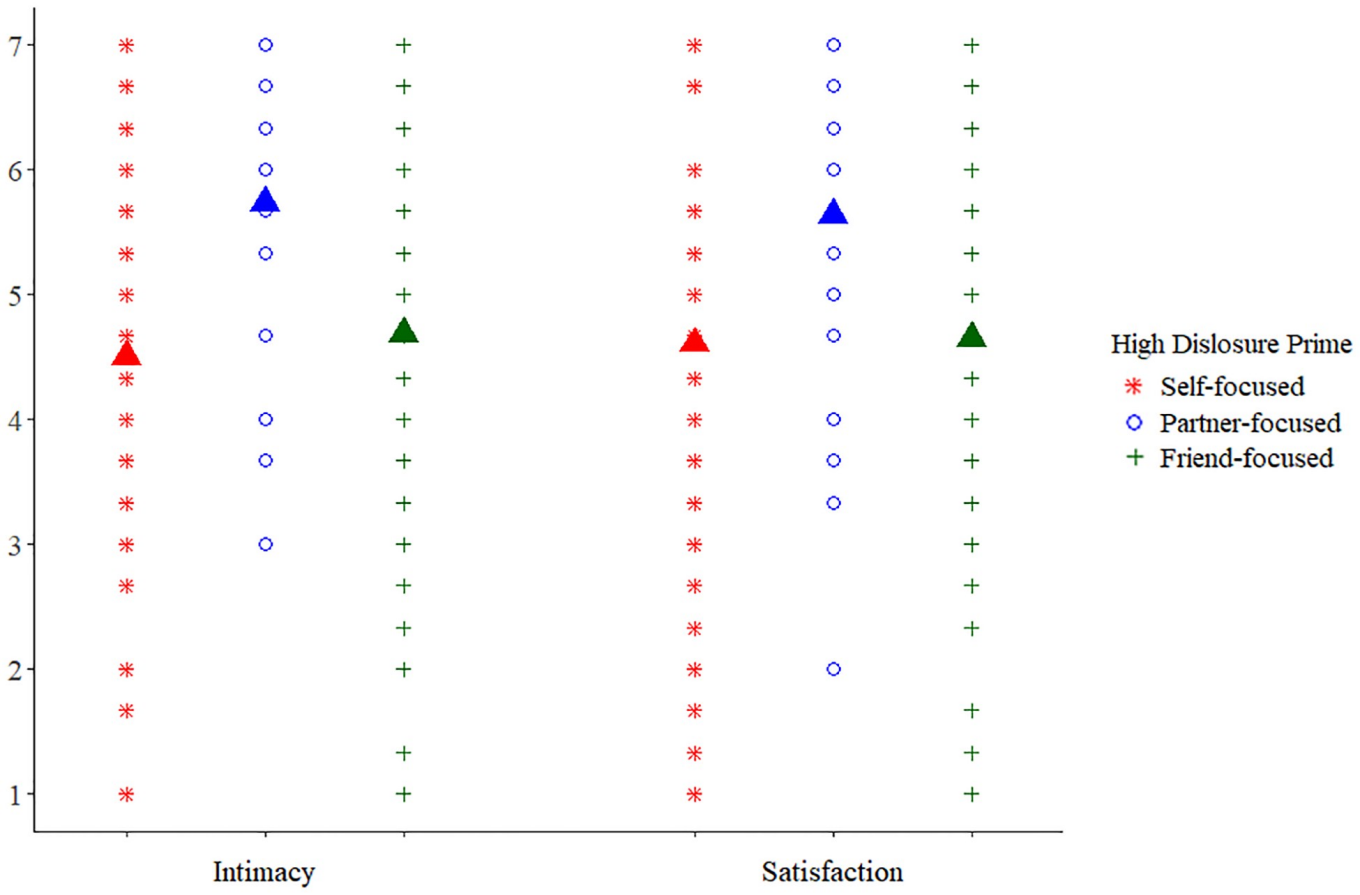
Study 5 means for intimacy and satisfaction in each of the prime conditions.

The ANCOVA for satisfaction revealed a similar pattern, with a main effect for prime type, *F*(2, 149) = 7.62, *p* = .001. Pairwise comparisons again using Fisher’s LSD test showed differences between the partner-focused prime (*M* = 5.64, *SD* = 1.11) and self-focused prime (*M* = 4.61, *SD* = 1.69), *p* = .001, and friend-focused prime (*M* = 4.63, *SD* = 1.61), *p* = .001, such that being exposed to the partner-focused prime led participants to report higher satisfaction compared to both other primes (see Fig 4 for scatterplot and means according to disclosure focus for intimacy and satisfaction). Offline disclosure also predicted satisfaction, in that higher offline disclosure was associated with greater satisfaction, *F*(1, 149) = 11.47, *p* = .001. No other effects were significant in both models (see Table 6). In other words, partner-focused high-depth disclosure was more beneficial to relationship intimacy and satisfaction compared to self- or friend-focused high-depth disclosure.

**Table 6:** Study 5 ANCOVA models predicting relationship intimacy and satisfaction.

	Intimacy					Satisfaction				
Predictor	*df*	*Error*	*F*	*p*	*η*_*p*_^*2*^	*df*	*Error*	*F*	*p*	*η*_*p*_^*2*^
**Main effects**										
**Prime**	2	149	10.28	< .001	.12	2	149	7.62	.001	.09
**Covariates**										
**Gender**	1	149	0.08	.78	.001	1	149	0.14	.71	.001
**Offline disclosure**	1	149	9.83	.002	.06	1	149	11.47	.001	.07
**Corrected Model**	4	149	8.33	< .001	.18	4	149	7.27	< .001	.16
***N***	156					156				

The results of Study 5 suggest that including the partner or the relationship in the online disclosure (operationalized as timeline photos and status updates) neutralizes or possibly counters these negative effects. In addition, there was no difference between the self- and friend- focused (*M* = 4.69, *SD* = 1.66 for intimacy; *M* = 4.63, *SD* = 1.61 for satisfaction) primes. In other words, rather than personal (self-focused) vs. relational (other-focused) online disclosure having disparate effects on romantic relationships, it seems the difference is in whether the online disclosure specifically involves the romantic relationship or not. This implies perceiving your partner to highly disclose online about his or her friendships, and possibly other relationships, do not have the same beneficial effects as witnessing him or her disclose about you or your relationship. Together, these results suggest that the content of disclosure also moderates the recipient’s interpretation of it, and it can interact with other contextual factors to influence the association between disclosure and relational outcomes.

## General discussion

In five studies, we provided evidence that online disclosure has different associations with relational outcomes compared to offline disclosure and identified an underlying mechanism that accounted for the differential effects. Study 1 demonstrated that high online disclosure predicted lower intimacy and satisfaction in the discloser’s romantic relationships (RQ1). This was not the case though in the discloser’s friendships (RQ2). In contrast, high offline disclosure predicted higher intimacy and satisfaction in both the discloser’s romantic and friend relationships, similar to past findings regarding offline disclosure. In addition, we were able to validate our newly developed self-report measure of online disclosure using objective ratings of people’s online disclosure. In Study 2, we found higher online self-disclosure was linked to lower intimacy and satisfaction in the discloser’s partner (RQ1), supporting theoretical models that propose one’s disclosure affects both people in a relationship [18–19] and suggesting online disclosure is also a relational phenomenon. In Study 3 we provided causal support for the association found in Study 2, showing that high online disclosure leads to lower intimacy and satisfaction in the discloser’s partner (RQ1). Importantly, Study 4 uncovered a mechanism underlying the effects of online disclosure: perceptions of the disclosure as high on inclusivity of recipients (RQ3). Finally, Study 5 identified a context, partner-focused content of disclosure, which negated the negative effects of online disclosure on romantic relationships (RQ4). The five studies described above are the first of their kind to systematically examine interactive effects of context on the associations between disclosure and relational outcomes, and to identify an underlying mechanism behind those effects. Together, our results suggest online disclosure, when done incautiously, can bring more harm than good to romantic relationships, from their development [51] to maintenance [49].

In the current paper we were able to resolve the inconsistency in the literature surrounding online disclosure and relational outcomes [29, 37]. By highlighting the effects of different contexts, we were able to identify when online disclosure negatively affected the relationship (Studies 1–4) and when this effect was negated and possibly countered (Study 5). Previous studies did not parse these separate contexts and thus resulted in different conclusions; our paper explains why this would have happened.

In addition, the current paper focuses on the effects of online disclosure, expanding existing literature on disclosure and relationships by applying it to a novel context. Most of the relationship research on disclosure focused on offline disclosure, and hence the conclusions were confined to the offline context and potentially inaccurate in other contexts. Indeed, we found that online disclosure negatively affected romantic relationships, which differs from most previous findings demonstrating the positive outcomes of offline disclosure. This points to a problem in the literature that has not yet been highlighted, that (a) the offline context is what could have driven previous findings on disclosure and relationships, and (b) we do not have much information on how contexts that are different from the offline context will affect the disclosure process. It is important to resolve this problem to theoretically increase our knowledge of the contextual factors affecting the disclosure process, and because new platforms are going to emerge and be used in people’s lives, and thus this knowledge will be highly relevant to the general population.

Our studies suggest the field of close relationship research should be updated to reflect the influence of recent and upcoming technologies. As mentioned in the introduction, there are currently many scholarly articles on online social networks in areas such as communication studies, information science, and computer science. However, not many papers explore how the online context is associated with psychological relational outcomes. As many relationships are now being formed, maintained, and even dissipated online [4, 94] researchers in psychology should look into how relational processes unfold and are affected by this different context. The current paper is the first that looks into the psychological effects that online disclosure depth has on relationships. The paper takes into account the promising literature on new telecommunications platforms and combines it with the large body of research on disclosure outcomes, in a way that can be more broadly used for relationship research and research in social and personality psychology in general. Based on our findings, we propose previous models of disclosure and relational outcomes [18–19] should be extended to include (a) more intricate considerations of contextual interactions on the disclosure process, and (b) underlying mechanisms of context effects.

### Adopting a multi-method approach

Throughout the five studies, we used a multi-method approach to identify the various contextual effects on the disclosure process in close relationships. First, the five studies used different methods of data collection, such as self-report measures (Studies 1–5), objective ratings made by independent judges (Study 1), and partner reports (Study 2). Obtaining data from multiple sources decreases the probability of making common method variance interpretations and enabled us to test different intriguing hypotheses [95]. Second, the studies were comprised of both correlational (Studies 1–2) and experimental (Studies 3–5) designs. In contrast to the experimental literature on offline disclosure and relational variables [43], no studies—to our knowledge—have previously manipulated perceptions of online disclosure and measured relational outcomes. Our experimental methods provided evidence to support both causality and directionality, establishing that changes in (perceptions of partner) online disclosure influence intimacy and satisfaction, instead of disclosure changing as a result of intimacy or satisfaction levels. Third, the studies addressed effects of disclosure on both the discloser (Study 1) and the discloser’s partner (Studies 2–5). Examining how both people involved in a relationship were influenced by the disclosure process enabled us to paint a more comprehensive picture on how contextual factors and disclosure affect relational outcomes. The results of our research support the notion that disclosure is a relational process, in that both parties are affected by its occurrence. Finally, the studies assessed the effects of both self-perceived (Studies 1–2) and partner-perceived (Studies 3–5) online disclosure. This is important because there could be discrepancies between self and partner perceptions of constructs [96]. We found that online disclosure, whether self- or partner-perceived, has negative implications for relational outcomes. This convergence of results suggests that the partner’s perception of one’s disclosure is more accurate than not. Using this approach allowed us to reinforce our findings and further detail the effects of contextual factors on the disclosure process in relationships. Although our findings on the conditional effects of disclosure are not exhaustive nor comprehensive, we had enough pieces to support the importance of context while providing meaningful examples.

### Effects of contexts and their interactions

In the five studies reported above, we found negative effects of online disclosure on relational outcomes (intimacy and satisfaction). However, the differential effects of disclosure type (online vs. offline) additionally depended on other contextual factors. First, the negative effects of online disclosure were only present in romantic relationships and not in friendships. Relationship type likely affects the associations between disclosure and relational processes and outcomes by influencing people’s expectations, perceptions, and beliefs surrounding the disclosure. These changes in expectations, perceptions, and beliefs can affect the interpretation of the disclosure and in turn the valence and strength of disclosure’s associations with relational outcomes. Such interpretation is more likely to have negative outcomes in relationships which harbor expectations about feeling special and exclusivity, such as romantic relationships [61–62].

Second, changing the focus of the disclosure content to the romantic relationship rather than the self or other relationships dissipated the negative effect of online disclosure on romantic relationships. This might be because disclosing about the self in a public space, such as an online social network, without including information about the romantic partner or relationship may lead the partner to feel excluded or left out. Studies have shown that perceptions of partner exclusion is associated with lower relationship well-being [90], similar to studies about general exclusion on ostracism [97]. Conversely, disclosure about the relationship is likely interpreted by the partner as inclusive and caring, and validates the relationship and the partner, which tends to be beneficial to the relationships [98]. Such disclosure, in turn, is likely to result in relatively heightened intimacy and satisfaction. Moreover, although we tested one kind of disclosure content, other contents may further yield different results. For example, if one partner discloses a secret about their relationship on Facebook, online disclosure may affect intimacy and satisfaction negatively even if the disclosure is about the partner and/or the relationship.

Delineating the ways that various contextual interactions affect the association between disclosure and relational outcomes is important in order to depict a comprehensive account of the disclosure process. It is also important because technological advances result in new disclosure mediums being introduced at a staggering rate. In the current paper we focused only on one type of online disclosure in comparison to offline disclosure: that being done via online social networks to a broad audience. More specifically, we have focused on the audience or recipient inclusivity of online mediums and how that affects the disclosure process. Through this operationalization, online disclosure is similar to generalized disclosure (conceptualized as disclosing to multiple others in one setting) and offline disclosure is similar to dyadic disclosure (conceptualized as disclosing to one person in one setting). Importantly, before the proliferation of online social networks, it was uncommon for high-depth generalized disclosure to occur, which is why the effects of high-depth generalized disclosure vs. high-depth dyadic disclosure have not been compared up until now. However, emerging platforms are enabling people to disclose online in other ways, which are even more removed from offline dyadic disclosure. For example, people can easily store online messages in their cellphones and look back at them whenever convenient [99], which may facilitate continuous rumination regarding past disclosures. When sending online messages, several platforms enable people to see whether or not the other person is typing a message, and they can even see when the person stops and decides not to send one [100]. These platforms have the potential to change the nature of disclosure itself, in that it may qualitatively differ from offline disclosure. Furthermore, the context of disclosure can encompass other situational circumstances such as ecological affordances for mobility, relationship developmental stage, point in life course, and relationship type, all which might have differential influences on the association between disclosure and relational outcomes.

### Underlying mechanisms of context effects

Our paper shows the first study that uncovered a mechanism underlying the effects of online disclosure depth on romantic relationships—level of inclusivity of recipients. By identifying a mechanism, we are able to explain how specific parts of previous models are affected by the online setting and more accurately update them. As previously mentioned, one way a mechanism can affect relational outcomes is by changing the interpretation of the disclosure. Specifically, in the person-situation interaction model [18] the interpretation of a message is based on various expectations and attributions within the partner. Similarly, in the interpersonal process model [19], the partner’s interpretive filter is theorized to be influenced by expectations and schemas. In the case of highly inclusive disclosure, people want to have a special role as the disclosure recipient rather than being one out of many [82], and this yearning is likely heightened in romantic relationships. Seeing one’s partner disclosing online may make a person interpret or feel as if the self is one of many, rather than special or unique. This may cause one to feel devalued [101] and negatively affect the relationship.

Likewise, there are possible other mechanisms that can affect various stages in the disclosure process [19]. For example, the high ambiguity of disclosure recipients and the resulting jealousy felt by the partner may also account for negative effects of online disclosure on romantic relationships by affecting the interpretation or interpretive filter. The *ambiguity of recipients* refers to the degree of certainty in determining who the recipients of the message are. In disclosure via online social networks, the recipients of the message tend to be highly ambiguous: Out of the many people connected to the discloser, the discloser does not know who (1) has actually received the message, and (2) who out of the recipients actually read the message [37]. This kind of ambiguous jealousy-evoking situation [37, 102] may increase suspicion and in turn affect the partner’s interpretive filter in a negative way, decreasing intimacy. Jealousy has also been shown to associate with lower satisfaction in romantic couples [103–104], which may explain our findings about online disclosure and satisfaction.

In addition to the discloser, recipients of online disclosure also tend to show lower *responsiveness* [15, 105]. This may happen because not everyone to whom the message is sent receives it, and those who do receive it, not all respond, due to factors such as lack of interest, time constraints, or processes such as diffusion of responsibility [106–107]. Indeed, people were found to respond only to a small part of the information they are exposed to online [108]. The discloser, however, may interpret this lack of response as disinterest or neglect, which may lower his or her relational intimacy and satisfaction. Future studies should test these proposed additional mechanisms.

#### Extended model of the disclosure process

**Fig 5:**
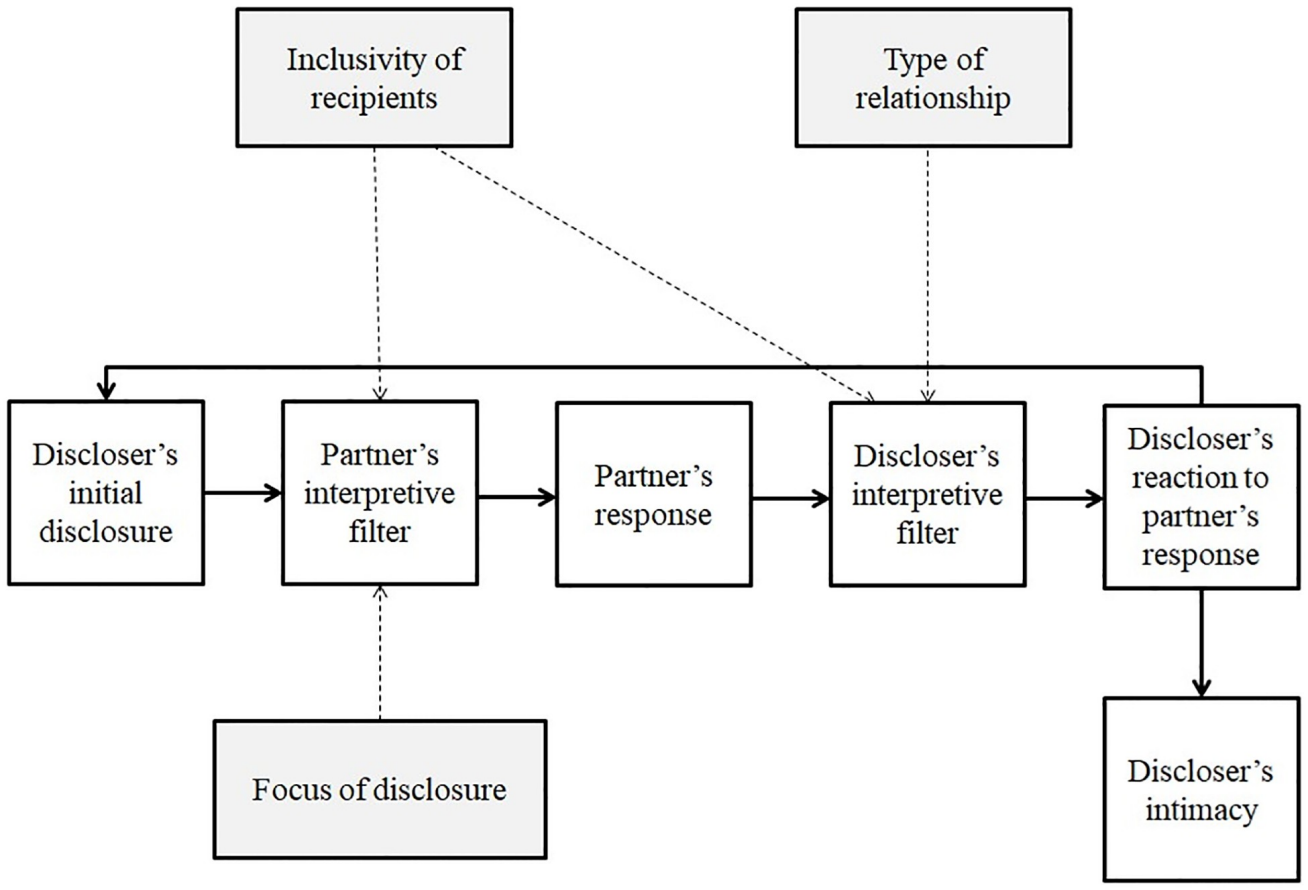
Extended interpersonal process model.

Based on our findings, we propose that previous models of disclosure should be extended to include detail on how contextual factors and underlying mechanisms may affect the disclosure process. We used the interpersonal process model as an illustration because that model incorporates both the discloser and partner to a greater degree than the person-situation interactional model. Fig 5 shows an example of the extended interpersonal process model [19] based on the findings of our five studies. The inclusivity of recipients (higher in online vs. offline) affects the partner’s (Studies 2–5) and discloser’s (Study 1) interpretive filter, the type of relationship influences the discloser’s (Study 1) interpretive filter, and the focus of disclosure affects the partner’s (Study 5) interpretive filter. It is highly likely that the three contexts we tested affect other stages of the disclosure process as well, and there are potentially many more contexts that we have not examined here. Let us clarify that we are using this model as an example to integrate our studies, and that in our studies we have only tested portions of the model. Future studies should endeavor more complete updates of the model. Extending previous models of disclosure is important because (a) the world is changing and many models are out of date; and (b) such updates would provide a theory-based framework that integrates the effects of context on relational processes. This may help not only scholars but also practitioners in developing better interventions to promote relational health.

### Limitations and future directions

There are a few limitations to the current studies. We focused on using Facebook to test our hypotheses about the effects of public online disclosure. Although Facebook is the most widely used online social networking site [109], and Facebook offers different forms of disclosure such as public ‘walls/timelines’ or more private messages, we acknowledge there are many more ways to disclose online, such as post on public online forums, send e-mails, texts, etc. Future studies should examine whether our findings hold in other forms of online disclosure. This is important because the Facebook online disclosure we assessed here and the offline dyadic disclosure we compared it to, have many more differences than merely being online and offline. For example, disclosure on Facebook typically occurs in a group setting, whereas offline disclosure often occurs in a one-to-one setting. In addition, as we showed, disclosure on Facebook is high on inclusivity, whereas offline dyadic disclosure is more exclusive. Finally, in Facebook, the responses that are being made to disclosures tend to convey less information and are likely to be less clear. For example, people provide fewer nonverbal cues in online compared to offline interactions, such as body language and intonation, which can make the message harder to interpret [110–111]. These differences may also account for the different relational outcomes compared with offline disclosure. Future studies may test the effect of only the online vs. offline context in disclosure by bringing existing dyads (friends and/or romantic partners) into the laboratory and randomly assigning them to disclose to each other either online or offline (and additionally with or without the presence of others), then measure any enhanced intimacy and satisfaction.

Although we have assumed participants would report their amount of offline disclosure in response to the measure, Miller’s scale does not specify “offline” or “face-to-face” disclosure in its instructions. Rather, it may reflect a more general disposition of participants to disclose to general others. To control for that, future studies should ask participants to report the amount of offline disclosure done specifically to the partner. As our focus here was mainly on online disclosure, we did not further examine these issues.

Our research was also based on the assumption that the partner would be aware of/subjected to the discloser’s online disclosure. If one’s romantic partner does not use online social network sites such as Facebook, it is possible that online disclosure would have less of an impact on the relationship than was found in our studies. Future studies might want to include a question about such exposure.

A limitation of Study 2 is that we were unable to use a multivariate model to account for the interdependence of the participant and partner data due to lacking parallel variables. This is because the focus of our study was not on interdependence in the relationship. Nevertheless, it would be interesting to see if our findings are replicated while accounting for interdependence in the relationship by modeling both actor and partner effects in future research.

We would like to note that the negative effects of high online personal disclosure on romantic relationships may not apply to long-distance relationships. Long-distance relationships are different from geographically close relationships in that the involved parties do not spend as much time face-to-face [112], and try to compensate by purposely disclosing more to their partner [113]. In the context of long-distance relationships, high online disclosure may have positive rather than negative effects. In support of this idea, in Study 4, when we took out those in long distance relationships (*N* = 9), we had a stronger effect of the relational prime on both intimacy and satisfaction. This shows that the type of romantic relationship (whether long-distance or not) is likely to matter for the effects of online disclosure.

We would also like the reader to note that we have grouped together several modes of self-disclosure (e.g., photos, text/status updates, profile information), as well as several venues of self-disclosure (e.g., on the profile, through status updates, through private messages) in our studies. Although we chose this method in order to increase the external validity of our primes, as online disclosure is a complicated phenomenon, doing so may increase the risk that our results become less discriminate. For instance, the items in our online self-disclosure scale pertain to many different modes, such as profile information, photos, links, and status updates. We would like the reader to keep in mind when interpreting our results is that different operationalizations were used throughout. For instance, Studies 1 and 2 are about general profile disclosures, very broadly speaking. Studies 3 and 5 are specifically about timeline photos and status updates, whereas Study 4 focuses on private messages as the context. Despite that, our results provide a consistent and coherent pattern, suggesting that grouping them together was not that bad of a decision to begin with. As mentioned above, we specifically used different types of disclosure (profile, status updates, messages) to account for the inherent variation that exists in current online social network (e.g, Facebook)-style disclosure. In other words, although interpretation of each study is a little different, this is as much a strength of the studies as a limitation, because just focusing on one of those contexts wouldn’t be generalizable to actual online disclosure.

Furthermore, although we tested the causal effect of online disclosure on intimacy/satisfaction in their experimental studies, it does not negate the possibility that the other direction is also significant. There can still be a bidirectional association between online disclosure and intimacy/satisfaction, which future research should explore.

## Conclusion

Most current research on disclosure and romantic relationships focuses on offline disclosure and emphasizes the positive outcomes of disclosure. The upsurge of digital technology and new affordances for communication necessitates a paradigm shift. In five studies we showed that online disclosure has the potential to actually hamper relationships—both their development and maintenance. Importantly, we identified a mechanism accounting for that effect–the high inclusivity of recipients. More broadly we have demonstrated that disclosure’s relational outcomes depend on the interplay of contexts, and highlighted the need to take the context (e.g., online new technologies) into consideration when reading existing literature or interpreting new findings. Future research on disclosure and close relationships should focus on identifying the specific mechanisms that lead to relational outcomes in order for people to maximize the benefits of new technology rather than fall victim to its poisons.

## Supporting information

S1 Appendix Online (Facebook and Twitter) self-disclosure scale. (DOCX)

S2 Appendix Study 4 low disclosure and high disclosure messages with inclusivity prime instructions. (DOCX)

S1 Table Study 1 Zero-Order correlations, means, and standard deviations for variables. (DOCX)

S2 Table Study 2 Zero-Order correlations, means, and standard deviations for variables. (DOCX)

S3 Table Study 3 Zero-Order correlations, means, and standard deviations for variables. (DOCX)

S4 Table Study 3 prime pretest analysis results. (DOCX)

S5 Table Study 4 Zero-Order correlations, means, and standard deviations for variables. (DOCX)

S6 Table Study 4 message pretest analysis results. (DOCX)

S7 Table Study 5 Zero-Order correlations, means, and standard deviations for variables. (DOCX)

S8 Table Study 5 prime pretest analysis results. (DOCX)

S1 Text (DOCX)

S2 Text (DOCX)
