# Supplementary material for: Effects of self- and partner’s online disclosure on relationship intimacy and satisfaction
Source: PLoS One. 2019 Mar 4;14(3):e0212186. doi: 10.1371/journal.pone.0212186 (PMC6398828; doi:10.1371/journal.pone.0212186)
Supplement: S1 Appendix — (DOCX) [file pone.0212186.s001.docx]

**S1 Appendix. Online (Facebook and Twitter) Self-disclosure Scale.**

Think of your use of social networking sites such as Facebook and Twitter. Then rate the following items on how often they pertain to you. Write the number in the space provided, using the following rating scale:

| **1** | **2** | **3** | **4** | **5** | **6** | **7** |
| --- | --- | --- | --- | --- | --- | --- |
| ***Not true of***  ***me at all*** | ***.........*** | ***.........*** | ***Neutral/***  ***Mixed*** | ***.........*** | ***.........*** | ***Definitely true***  ***of me*** |

_____ 1. I believe another Facebook user can get a pretty good sense of who I am after simply viewing the information I have disclosed on my Facebook profile.

_____ 2. I believe another Facebook user would have a difficult time knowing who I am after simply viewing the information I have disclosed on my Facebook profile. (Reverse-scored)

_____ 3. I frequently post pictures and links that summarize the events of my daily life.

_____ 4. When I update my Facebook or Twitter “status”, I tend to post something that displays the emotional state I am currently in.

_____ 5. When I update my Facebook or Twitter “status”, I tend to post my opinion of something that my Facebook friends may find relevant.

_____ 6. When I update my Facebook or Twitter “status”, I am doing so to express myself to anyone that may be interested.

_____ 7. When I update my Facebook or Twitter “status”, I am not afraid to post something I consider personal.

_____ 8. When I update my Facebook or Twitter “status”, I try to stay away from anything I consider at all personal. (Reverse-scored)
