# Supplementary material for: Effects of self- and partner’s online disclosure on relationship intimacy and satisfaction
Source: PLoS One. 2019 Mar 4;14(3):e0212186. doi: 10.1371/journal.pone.0212186 (PMC6398828; doi:10.1371/journal.pone.0212186)
Supplement: S2 Appendix — (DOCX) [file pone.0212186.s002.docx]

**S2 Appendix. Study 4 Low Disclosure and High Disclosure Messages with Inclusivity Prime Instructions.**

**Low Disclosure Message**

Message 1:

"3 hours of training at work...it took a long time. Lots of information to take in, but its all new to me."

Message 2:

"Let's go team! Great win tonight!"

**High Disclosure Message**

Message 1:

"3 hours of training at work...pretty interesting. I'm learning a bunch of new things that we never expected to. If you wanna see something scary google electronic accidents"

Message 2:

"Had a real nice night tonight! You guys are awesome!"

**1 Recipient Instructions**

Imagine that you have received the following Facebook messages from your partner. The messages have been sent only to you.

**2 Recipient Instructions**

Imagine that you have received the following Facebook messages from your partner. The messages have been sent to you and one other person.

**5 Recipient Instructions**

Imagine that you have received the following Facebook messages from your partner. The messages have been sent to you and four other people.

**20 Recipient Instructions**

Imagine that you have received the following Facebook messages from your partner. The messages have been sent to you and 19 other people.

**25 Recipient Instructions**

Imagine that you have received the following Facebook messages from your partner. The messages have been sent to you and 24 other people.
