# Supplementary material for: Effects of self- and partner’s online disclosure on relationship intimacy and satisfaction
Source: PLoS One. 2019 Mar 4;14(3):e0212186. doi: 10.1371/journal.pone.0212186 (PMC6398828; doi:10.1371/journal.pone.0212186)
Supplement: S1 Table — (DOCX) [file pone.0212186.s003.docx]

**S1 Table.** **Study 1 Zero-Order Correlations, Means, and Standard Deviations for Variables.**

|  | Variable | *1* | *2* | *3* | *4* | *5* | *6* | *7* | Range | *M* ± *SD* |
| --- | --- | --- | --- | --- | --- | --- | --- | --- | --- | --- |
| 1. | Gender | — |  |  |  |  |  |  | N/A | N/A |
| 2. | Offline self-disclosure | .28*** | — |  |  |  |  |  | 1–7 | 5.05 ± 1.16 |
| 3. | Online self-disclosure | .15* | .12 | — |  |  |  |  | 1–6 | 3.37 ± 1.02 |
| 4. | Intimacy with partner | .09 | .30** | -.16 | — |  |  |  | 2.67–7 | 5.96 ± 1.13 |
| 5. | Satisfaction with partner | .13 | .30** | -.26** | .76*** | — |  |  | 2–7 | 5.79 ± 1.27 |
| 6. | Intimacy with friend | .25*** | .36*** | .03 | .39*** | .41*** | — |  | 1–7 | 5.27 ± 1.28 |
| 7. | Satisfaction with friend | .14 | .39*** | -.04 | .32** | .47*** | .69*** | — | 1–7 | 5.68 ± 1.23 |

*Note*. Gender was coded as men = 1 and women = 2.

*Note 2*. * *p* < .05, ** *p* < .01, *** *p* < .001.
