# Supplementary material for: Effects of self- and partner’s online disclosure on relationship intimacy and satisfaction
Source: PLoS One. 2019 Mar 4;14(3):e0212186. doi: 10.1371/journal.pone.0212186 (PMC6398828; doi:10.1371/journal.pone.0212186)
Supplement: S2 Table — (DOCX) [file pone.0212186.s004.docx]

**S2 Table.** **Study 2 Zero-Order Correlations, Means, and Standard Deviations for Variables.**

|  | Variable | *1* | *2* | *3* | *4* | *5* | *6* | *7* | Range | *M* ± *SD* |
| --- | --- | --- | --- | --- | --- | --- | --- | --- | --- | --- |
| 1. | Gender | — |  |  |  |  |  |  | N/A | N/A |
| 2. | Attachment anxiety | .25* | — |  |  |  |  |  | 1.53–6.78 | 3.56 ± 1.16 |
| 3. | Attachment avoidance | .04 | .38** | — |  |  |  |  | 1–4.72 | 2.77 ± 0.91 |
| 4. | Offline self-disclosure | .22 | .04 | -.26* | — |  |  |  | 3.55–7 | 5.51 ± 0.90 |
| 5. | Online self-disclosure | -.09 | .12 | -.05 | .43*** | — |  |  | 1.38–5.5 | 3.37 ± 0.96 |
| 6. | Intimacy of partner | .02 | .08 | -.35** | .13 | -.22 | — |  | 4.33–7 | 6.35 ± 0.71 |
| 7. | Satisfaction of partner | -.04 | -.10 | -.29* | .18 | -.14 | .67*** | — | 4.33–7 | 6.45 ± 0.70 |

*Note*. Gender was coded as men = 1 and women = 2.

*Note 2*. * *p* < .05, ** *p* < .01, *** *p* < .001.
