# Supplementary material for: Effects of self- and partner’s online disclosure on relationship intimacy and satisfaction
Source: PLoS One. 2019 Mar 4;14(3):e0212186. doi: 10.1371/journal.pone.0212186 (PMC6398828; doi:10.1371/journal.pone.0212186)
Supplement: S4 Table — (DOCX) [file pone.0212186.s006.docx]

**S4 Table. Study 3 Prime Pretest Analysis Results.**

|  | Low disclosure  (*N* = 13) |  | High disclosure  (*N* = 18) |  | *t*(29) |  | *p* |  | Cohen’s *d* |
| --- | --- | --- | --- | --- | --- | --- | --- | --- | --- |
| Question | Mean (SD) |  | Mean (SD) |  |  |  |  |  |  |
| What do you think is the age of the 'wall' owner? | 21.31 (0.75) |  | 21.44 (1.04) |  | -0.40 |  | .69 |  | -0.15 |
| After seeing the 'wall,' how appealing does its owner look to you? | 3.46 (1.20) |  | 3.22 (1.31) |  | 0.52 |  | .80 |  | 0.19 |
| After seeing the 'wall,' think about its potential owner and tell us how interested you think you’ll be in forming a long-term romantic relationship with him/her? | 2.08 (1.32) |  | 2.44 (1.20) |  | -0.81 |  | .43 |  | -0.30 |
| After seeing the 'wall,' how much do you think you'd like its owner? | 3.77 (1.30) |  | 3.67 (1.46) |  | 0.20 |  | .84 |  | 0.07 |
| On a scale of 1 (not at all) to 7 (very much), how obnoxious do you think the owner of this 'wall' is? | 3.77 (1.79) |  | 3.50 (1.42) |  | 0.47 |  | .64 |  | 0.17 |
| After seeing this 'wall,' how warm/cold does its owner look to you? On a 1 (very cold) to 7 (very warm) scale? | 4.77 (0.83) |  | 4.33 (1.14) |  | 1.17 |  | .25 |  | 0.43 |
| On a scale of 1 (not at all) to 7 (very much), how much do you think this person is self-disclosing? | 3.46 (1.13) |  | 4.72 (1.36) |  | -2.73 |  | .01 |  | -1.01 |
| How much information do you feel this 'wall' contains? | 3.46 (1.45) |  | 3.72 (1.36) |  | -0.51 |  | .61 |  | -0.20 |
| After seeing the 'wall,' how would you rate your positive mood right now? Please use the following scale, from 1 (not positive at all) to 7 (very positive) scale? | 4.62 (0.65) |  | 4.22 (0.81) |  | 1.45 |  | .16 |  | 0.54 |
| After seeing the 'wall,' how would you rate your negative mood right now? Please use the following scale, from 1 (not negative at all) to 7 (very negative) scale? | 2.85 (1.14) |  | 2.83 (1.47) |  | 0.03 |  | .98 |  | 0.01 |
| How appealing does this 'wall' look to you? | 3.23 (1.36) |  | 3.11 (1.23) |  | 0.26 |  | .80 |  | 0.10 |
| On a scale of 1 (not at all) to 7 (very much), how interesting is the content of this 'wall'? | 3.31 (1.25) |  | 2.89 (1.32) |  | 0.89 |  | .38 |  | 0.33 |
| How aesthetically pleasing does this 'wall' look to you? | 3.39 (1.45) |  | 3.39 (1.42) |  | -0.01 |  | .99 |  | -0.004 |
|  | Low disclosure  (*N* = 18) |  | High disclosure  (*N* = 13) |  | *χ^2^*(3, *N* = 31) |  | *p* |  | *ϕ* |
| Question | Count |  | Count |  |  |  |  |  |  |
| What do you think is the gender of the 'wall' owner? | “Male” = 11, “Female” = 1,  “Could be either” = 1,  “Couldn’t tell” = 0 |  | “Male” = 8, “Female” = 5,  “Could be either” = 4,  “Couldn’t tell” = 1 |  | 5.27 |  | .15 |  | .41 |
