# Supplementary material for: Effects of self- and partner’s online disclosure on relationship intimacy and satisfaction
Source: PLoS One. 2019 Mar 4;14(3):e0212186. doi: 10.1371/journal.pone.0212186 (PMC6398828; doi:10.1371/journal.pone.0212186)
Supplement: S5 Table — (DOCX) [file pone.0212186.s007.docx]

**S5 Table. Study 4 Zero-Order Correlations, Means, and Standard Deviations for Variables.**

|  | Variable | *1* | *2* | *3* | *4* | Range | *M* ± *SD* |
| --- | --- | --- | --- | --- | --- | --- | --- |
| 1. | Gender | — |  |  |  | N/A | N/A |
| 2. | Offline self-disclosure | .12 | — |  |  | 0.8–4 | 2.90 ± 0.78 |
| 3. | Intimacy with partner | .11 | .41*** | — |  | 1–7 | 5.26 ± 1.56 |
| 4. | Satisfaction with partner | .05 | .41*** | .90*** | — | 1–7 | 5.29 ± 1.57 |

*Note*. Gender was coded as men = 1 and women = 2.

*Note 2*. Intimacy and satisfaction with partner variables are collapsed across experimental conditions.

*Note 3*. * *p* < .05, ** *p* < .01, *** *p* < .001.
