# Supplementary material for: Effects of self- and partner’s online disclosure on relationship intimacy and satisfaction
Source: PLoS One. 2019 Mar 4;14(3):e0212186. doi: 10.1371/journal.pone.0212186 (PMC6398828; doi:10.1371/journal.pone.0212186)
Supplement: S6 Table — (DOCX) [file pone.0212186.s008.docx]

**S6 Table. Study 4 Message Pretest Analysis Results.**

|  | Low disclosure  (*N* = 10) |  | High disclosure  (*N* = 10) |  | *t*(18) |  | *p* |  | Cohen’s *d* |
| --- | --- | --- | --- | --- | --- | --- | --- | --- | --- |
| Question | Mean (SD) |  | Mean (SD) |  |  |  |  |  |  |
| Think about the message's sender and tell us how interested you think you’ll be in forming a long-term romantic relationship with him/her? | 1.70 (1.60) |  | 2.10 (1.20) |  | -0.73 |  | .48 |  | -0.34 |
| After reading the message, how much do you think you'd like the sender? | 3.80 (1.14) |  | 4.40 (0.84) |  | -1.34 |  | .20 |  | -0.63 |
| On a scale of 1 (not at all) to 7 (very much), how obnoxious do you think the sender of the message is? | 2.70 (1.89) |  | 3.40 (1.78) |  | -0.85 |  | .40 |  | -0.40 |
| On a scale of 1 (not at all) to 7 (very much), how much do you think the sender of the message is self-disclosing? | 2.90 (1.60) |  | 4.80 (1.14) |  | -3.07 |  | .007 |  | -1.45 |
| How much information do you feel this message contains? | 2.70 (1.89) |  | 3.60 (1.51) |  | -1.60 |  | .13 |  | -0.75 |
|  | Low disclosure  (*N* = 10) |  | High disclosure  (*N* = 10) |  | *χ^2^*(3, *N* = 20) |  | *p* |  | *ϕ* |
| Question | Count |  | Count |  |  |  |  |  |  |
| What do you think is the gender of the message sender? | “Male” = 4, “Female” = 2,  “Could be either” = 3,  “Couldn’t tell” = 1 |  | “Male” = 4, “Female” = 4,  “Could be either” = 2,  “Couldn’t tell” = 0 |  | 1.87 |  | .60 |  | .31 |
