# Supplementary material for: Effects of self- and partner’s online disclosure on relationship intimacy and satisfaction
Source: PLoS One. 2019 Mar 4;14(3):e0212186. doi: 10.1371/journal.pone.0212186 (PMC6398828; doi:10.1371/journal.pone.0212186)
Supplement: S7 Table — (DOCX) [file pone.0212186.s009.docx]

**S7 Table. Study 5 Zero-Order Correlations, Means, and Standard Deviations for Variables.**

|  | Variable | *1* | *2* | *3* | *4* | Range | *M* ± *SD* |
| --- | --- | --- | --- | --- | --- | --- | --- |
| 1. | Gender | — |  |  |  | N/A | N/A |
| 2. | Offline self-disclosure | .33*** | — |  |  | 0–4 | 2.93 ± 0.85 |
| 3. | Intimacy with partner | .07 | .26*** | — |  | 1–7 | 4.97 ± 1.54 |
| 4. | Satisfaction with partner | .07 | .28*** | .91*** | — | 1–7 | 4.95± 1.55 |

*Note*. Gender was coded as men = 1 and women = 2.

*Note 2*. Intimacy and satisfaction with partner variables are collapsed across experimental conditions.

*Note 3*. * *p* < .05, ** *p* < .01, *** *p* < .001.
