# Supplementary material for: Effects of self- and partner’s online disclosure on relationship intimacy and satisfaction
Source: PLoS One. 2019 Mar 4;14(3):e0212186. doi: 10.1371/journal.pone.0212186 (PMC6398828; doi:10.1371/journal.pone.0212186)
Supplement: S8 Table — (DOCX) [file pone.0212186.s010.docx]

**S8 Table. Study 5 Prime Pretest Analysis Results.**

|  | Self-focused high disclosure  (*N* = 10) |  | Partner-focused high disclosure  (*N* = 11) |  | Friend-focused high disclosure  (*N* = 11) |  | *F*(2, 29) | |  | *p* |  | *η^2^* |
| --- | --- | --- | --- | --- | --- | --- | --- | --- | --- | --- | --- | --- |
| Question | Mean (SD) |  | Mean (SD) |  | Mean (SD) |  |  | |  |  |  |  |
| What do you think is the age of the 'wall' owner? | 22.60 (2.01) |  | 21.18 (1.89) |  | 23.36 (2.69) |  | 1.34 | |  | .28 |  | .08 |
| After seeing the 'wall,' how appealing does its owner look to you? | 3.50 (0.84) |  | 3.55 (1.44) |  | 4.55 (1.13) | |  | 2.19 |  | .13 |  | .13 |
| After seeing the 'wall,' how much do you think you'd like its owner? | 3.80 (1.23) |  | 4.27 (2.00) |  | 4.27 (0.91) | |  | 0.36 |  | .70 |  | .02 |
| On a scale of 1 (not at all) to 7 (very much), how obnoxious do you think the owner of this 'wall' is? | 3.80 (1.40) |  | 2.91 (1.58) |  | 3.45 (1.70) | |  | 0.87 |  | .43 |  | .06 |
| After seeing this 'wall,' how warm/cold does its owner look to you? On a 1 (very cold) to 7 (very warm) scale? | 4.40 (1.08) |  | 5.18 (1.47) |  | 4.82 (0.75) | |  | 1.23 |  | .31 |  | .08 |
| On a scale of 1 (not at all) to 7 (very much), how much do you think this person is self-disclosing? | 4.20 (0.79) |  | 4.18 (0.98) |  | 3.73 (1.56) | |  | 0.57 |  | .57 |  | .04 |
| How much information do you feel this 'wall' contains? | 3.80 (1.32) |  | 3.64 (1.03) |  | 4.00 (1.27) | |  | 0.25 |  | .78 |  | .02 |
| After seeing the 'wall,' how would you rate your positive mood right now? Please use the following scale, from 1 (not positive at all) to 7 (very positive) scale? | 4.10 (1.20) |  | 4.45 (1.92) |  | 5.18 (0.75) | |  | 1.69 |  | .20 |  | .10 |
| After seeing the 'wall,' how would you rate your negative mood right now? Please use the following scale, from 1 (not negative at all) to 7 (very negative) scale? | 2.70 (1.34) |  | 2.91 (1.87) |  | 2.09 (1.30) | |  | 0.85 |  | .44 |  | .06 |
| How appealing does this 'wall' look to you? | 3.40 (0.84) |  | 3.55 (1.64) |  | 4.09 (1.14) |  | 0.89 | |  | .42 |  | .06 |
| On a scale of 1 (not at all) to 7 (very much), how interesting is the content of this 'wall'? | 3.20 (1.32) |  | 3.00 (1.48) |  | 3.27 (1.56) |  | 0.10 | |  | .90 |  | .01 |
| How aesthetically pleasing does this 'wall' look to you? | 3.30 (1.32) |  | 3.36 (1.57) |  | 4.64 (1.29) |  | 3.35 | |  | .05 |  | .19 |
| After seeing this 'wall,' to what extent do you think the owner's romantic partner would feel "left out"? | 3.90 (1.45) |  | 1.91 (0.94) |  | 4.45 (1.81) |  | 9.39 | |  | .001 |  | .39 |
| After seeing this 'wall,' to what extent do you think the owner's romantic partner is involved in the owner's life? | 2.80 (1.45) |  | 5.45 (1.57) |  | 2.36 (1.50) |  | 15.55 | |  | < .001 |  | .52 |
| How appropriate is the content on this 'wall'? | 4.70 (1.49) |  | 5.73 (1.56) |  | 5.73 (1.49) |  | 1.58 | |  | .22 |  | .10 |
| I find the posts on this 'wall' to be inappropriate. | 2.30 (1.49) |  | 1.73 (0.91) |  | 1.64 (1.03) |  | 1.00 | |  | .38 |  | .06 |
| I find the posts on this 'wall' to be too revealing. | 3.20 (1.69) |  | 1.91 (1.04) |  | 2.18 (1.40) |  | 2.47 | |  | .10 |  | .15 |
|  | Self-focused high disclosure  (*N* = 10) |  | Partner-focused high disclosure  (*N* = 11) |  | Friend-focused high disclosure  (*N* = 11) |  | *χ^2^*(6, *N* = 31) | |  | *p* |  | *ϕ* |
| Question | Count |  | Count |  | Count |  |  | |  |  |  |  |
| What do you think is the gender of the 'wall' owner? | “Male” = 9, “Female” = 1,  “Could be either” = 0,  “Couldn’t tell” = 0 |  | “Male” = 7, “Female” = 3,  “Could be either” = 1,  “Couldn’t tell” = 0 |  | “Male” = 7, “Female” = 2,  “Could be either” = 2,  “Couldn’t tell” = 0 |  | 5.46 | |  | .49 |  | .37 |
|  |  |  |  |  |  |  |  | |  |  |  |  |
