# Supplementary material for: Effects of self- and partner’s online disclosure on relationship intimacy and satisfaction
Source: PLoS One. 2019 Mar 4;14(3):e0212186. doi: 10.1371/journal.pone.0212186 (PMC6398828; doi:10.1371/journal.pone.0212186)
Supplement: S1 Text — (DOCX) [file pone.0212186.s011.docx]

**S1 Text.**

We collected relationship length only for Studies 2-5. In Study 2, we used it in an additional analysis where results showed the effect of online self-disclosure on intimacy (*Β* = -0.34, *t*(50) = -3.33, *p* = .002) and satisfaction (*Β* = -0.28, *t*(50) = -2.54, *p* = .01) remained significant when controlling for length. In Study 3, the effect of the disclosure prime on intimacy (*Β* = -0.69, *t*(122) = -2.48, *p* = .02) and satisfaction (*Β* = -0.78, *t*(122) = -2.99, *p* = .003) held when controlling for length. In Study 4, the interaction effect of the highest inclusivity prime and disclosure on intimacy (*Β* = -1.44, *t*(119) = -1.87, *p* = .065) became marginal, and satisfaction (*Β* = -1.78, *t*(119) = -2.30, *p* = .02) remained significant with length controlled. Finally, in Study 5, when controlling for length, the main effect of the prime on intimacy (*F*(2, 60) = 8.24, *p* = .001) and satisfaction (*F*(2, 60) = 5.18, *p* = .008) remained significant.
