# Supplementary material for: Effects of self- and partner’s online disclosure on relationship intimacy and satisfaction
Source: PLoS One. 2019 Mar 4;14(3):e0212186. doi: 10.1371/journal.pone.0212186 (PMC6398828; doi:10.1371/journal.pone.0212186)
Supplement: S2 Text — (DOCX) [file pone.0212186.s012.docx]

**S2 Text.**

In the prime pretest of Studies 3 and 5, the majority of participants thought the ‘wall’ owner was male. However, there were no gender effects in the results of the actual studies, which imply that even the male participants, who were supposed to imagine their female partners were the ‘wall’ owners, had no issue doing so.
